# Video versus direct laryngoscopy in critically ill patients: an updated systematic review and meta-analysis of randomized controlled trials

**DOI:** 10.1186/s13054-023-04727-9

**Published:** 2024-01-02

**Authors:** Beatriz Araújo, André Rivera, Suzany Martins, Renatha Abreu, Paula Cassa, Maicon Silva, Alice Gallo de Moraes

**Affiliations:** 1 Department of Medicine, Nove de Julho University, 90 Dom Jaime de Barros Câmara Avenue, São Bernardo do Campo, São Paulo Brazil; 2 Division of Pulmonary and Critical Care Medicine, Mayo Clinic, Rochester, MN USA

**Keywords:** Critical care, Intubation, Laryngoscopy, Meta-analysis, Video laryngoscopy

## Abstract

**Background:**

The utilization of video laryngoscopy (VL) has demonstrated superiority over direct laryngoscopy (DL) for intubation in surgical settings. However, its effectiveness in the intensive care unit and emergency department settings remains uncertain.

**Methods:**

We systematically searched PubMed, Embase, Cochrane, and ClinicalTrials.gov databases for randomized controlled trials (RCTs) comparing VL versus DL in critically ill patients. Critical setting was defined as emergency department and intensive care unit. This systematic review and meta-analysis followed Cochrane and PRISMA recommendations. R version 4.3.1 was used for statistical analysis and heterogeneity was examined with I^2^ statistics. All outcomes were submitted to random-effect models.

**Results:**

Our meta-analysis of 14 RCTs, compromising 3981 patients assigned to VL (*n* = 2002) or DL (*n* = 1979). Compared with DL, VL significantly increased successful intubations on the first attempt (RR 1.12; 95% CI 1.04–1.20; *p* < 0.01; *I*^2^ = 82%). Regarding adverse events, VL reduced the number of esophageal intubations (RR 0.44; 95% CI 0.24–0.80; *p* < 0.01; *I*^2^ = 0%) and incidence of aspiration episodes (RR 0.63; 95% CI 0.41–0.96; *p* = 0.03; *I*^2^ = 0%) compared to DL.

**Conclusion:**

VL is a more effective and safer strategy compared with DL for increasing successful intubations on the first attempt and reducing esophageal intubations in critically ill patients. Our findings support the routine use of VL in critically ill patients.

*Registration* CRD42023439685 https://www.crd.york.ac.uk/prospero/display_record.php?ID=CRD42023439685. Registered 6 July 2023.

**Supplementary Information:**

The online version contains supplementary material available at 10.1186/s13054-023-04727-9.

## Background

Tracheal intubation plays a crucial role in the management of critically ill patients’ airways. Approximately, 1.6 millions of patients undergo orotracheal intubations yearly in the US [1]. The number of direct laryngoscopy attempts during intubation has been associated with poor outcomes, including airway complications and hemodynamic instability [2]. Notably, initial intubation attempts fail in approximately 20% in emergency department (ED) and intensive care unit (ICU). [3–6]

The video laryngoscope (VL) has emerged as a promising alternative, offering enhanced visualization of airway structures. VL demonstrated superiority over the gold standard, direct laryngoscope (DL), in surgical scenarios [7]. Meanwhile, approximately 80% of the intubations performed in the ED and ICU worldwide are performed with a DL [2]. However, DL could be challenging due to several factors. These include limited mouth aperture and potential instability of cervical spine [8–10]. Despite this, there is an ongoing debate on the efficacy and safety of VL in critically ill patients [11, 12].

Previous meta-analyses showed no significant difference in successful intubation on the first attempt in critically ill patients [7, 13]. However, several randomized controlled trials (RCTs) have been published recently, including the Direct versus Video Laryngoscope (DEVICE) trial, the largest to date, showing promising results [12, 14, 15]. To shed light on this controversy, we performed an updated systematic review and meta-analysis of RCTs comparing VL versus DL in critically ill patients.

## Methods

The systematic review and meta-analysis were performed and reported following the Cochrane Collaboration Handbook for Systematic Reviews of Interventions and the Preferred Reporting Items for Systematic Reviews and Meta-Analysis (PRISMA) Statement guidelines (Additional file 1: Supplemental Methods 1, 2).[16, 17] The prospective meta-analysis protocol was registered at the International Prospective Register of Systematic Reviews (PROSPERO; CRD42023439685) on the 6 July 2023.

### Data source and search strategy

We systematically searched PubMed, Embase, Cochrane Library, and ClinicalTrials.gov from inception to June 23, 2023. The search terms used included ‘video’, ‘intubation’, and ‘laryngoscope’. The complete search strategy is provided in Additional 1: Supplemental Methods 3. Two authors (B.A. and S.L.) independently screened titles and abstracts and evaluated the articles in full for eligibility based on prespecified criteria. Discrepancies were resolved in a panel discussion with a third author (A.R.). Moreover, we used backward snowballing (i.e., review of references) to identify relevant texts from articles identified in the original search.

### Eligibility criteria

We considered studies eligible for inclusion if they (1) were RCTs; (2) directly compared VL versus DL; (3) enrolled critically ill patients (admitted to ED or ICU); (4) included adult patients; and (5) presented data regarding any of the prespecified efficacy and safety endpoints. The exclusion criteria were non-randomized studies, quasi-RCTs, cluster RCTs, studies that included patients younger than 16 years old or pregnant patients, studies centered on surgical scenarios, or conference abstracts.

### Data extraction

Four authors (B.A., S.M., P.C., and M.S.) independently extracted the data for each study using a standardized study form to determine: authors, clinical trial registration, enrollment period, study publication year, main exclusion criteria (Additional file 1: *Supplemental Methods 4*), sample size, follow-up period, endpoint definition, baseline patient characteristics, and operator’s characteristics. Any discrepancies were settled through a panel discussion with a fifth author (A.R.).

The definition of operators' experiences slightly varied among studies. To allow subgroup analysis based on this characteristic, we classified operators into two groups, experienced and inexperienced, following specific criteria outlined in Additional file 1: *Supplemental Methods 5*. Moreover, the Additional file 1: *Supplemental Methods 6* highlights how each study selected the device for the second intubation attempt. The classification of a difficult airway was made in accordance with either the study’s definition or the Mallampati 3/4 classification.

### Endpoints

Our primary efficacy endpoint was (1) successful intubation on the first attempt, as defined by each individual study. Other efficacy endpoints were (2) successful intubation on the second attempt, (3) Cormack Lehane (CL) laryngeal view grade I, and (4) CL laryngeal view grade I/II. Safety endpoints were (5) incidence of aspiration, (6) esophageal intubation, (7) cardiac arrest, (8) severe hypoxemia, (9) dental injury, and (10) all-cause mortality. Additional file 1: *Supplemental Methods 7* describes the endpoint definition of some outcomes.

We conducted prespecified subgroup analyses for the primary outcome. Studies were grouped based on the (1) VL brands and (2) operators’ experience. A sensitivity analysis of the subgroup analysis evaluating the operator’s experience was performed changing the threshold of from 50 to 100 prior intubations to be considered experienced. Subgroup analyses were performed if two or more studies were available in the group.

### Quality assessment

Two independent authors (B.A. and R.A.) assessed the risk of bias in the included RCTs using Cochrane’s Collaboration tool for assessing the risk of bias in randomized trials (RoB 2) [18]. Any disagreements were resolved through consensus between authors. We explored the potential for publication bias by visual inspection of the comparison-adjusted funnel plots and Egger’s test for the primary endpoint.

### Statistical analysis

We used the random-effects model for all outcomes. We employed risk ratios (RRs) and 95% confidence intervals (CIs) as the measure of effect size for binary endpoints. For continuous endpoints, we utilized weighted mean differences (MDs). Restricted maximum likelihood estimator was used to calculate heterogeneity variance *t*^2^. We assessed heterogeneity with Cochrane’s *Q* statistic and Higgins and Thompson’s *I*^2^ statistic, with *p* ≤ 0.10 indicating statistical significance. We determined the consistency of the studies based on *I*^2^ values of 0%, ≤ 25%, ≤ 50%, and > 50%, indicating no observed, low, moderate, and substantial heterogeneity, respectively. All tests were two-tailed, and a *p* value of < 0.05 was considered statistically significant. If necessary, means and standard deviations were estimated [19]. We conducted a trial sequential analysis (TSA) using random-effects model for the primary outcome, we used a statistical significance level of 5% and a beta of 80%. We used TSA version 0.9.5.10 beta (Copenhagen Trial Unit, Centre for Clinical Intervention Research, Rigshospitalet, Copenhagen, Denmark). We used R version 4.3.1 and the extension packages "meta," "metafor", "dmetar", "ggplot2", and "forestplot" for all calculations and graphics [20–23]. An in-depth description of the statistical analyses is available in Additional 1: Supplemental Methods 8.

## Results

### Study selection and characteristics

**Fig. 1:**
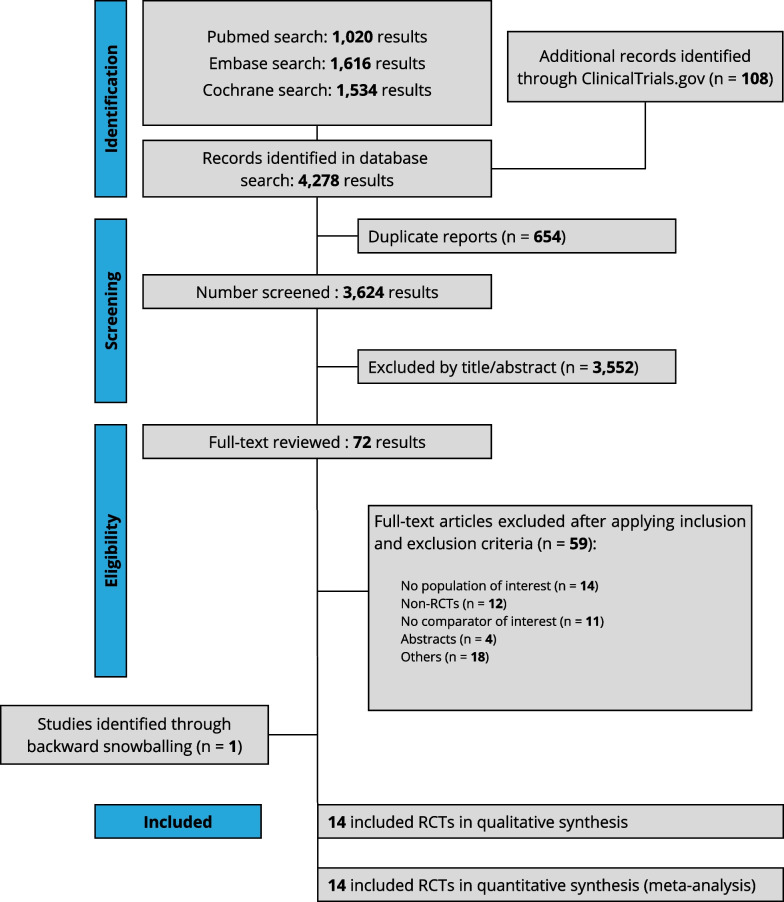
PRISMA flow diagram of study screening and selection. *Abbreviations:* RCT, randomized controlled trial

Our systematic search yielded 4278 potential articles (Fig. 1). After removing duplicates, 72 articles were retrieved and reviewed in full for possible inclusion. Of these, 14 RCTs met all inclusion criteria and were included in the primary analysis [11, 12, 14, 15, 24–32]. We included a total of 3981 patients, of whom 2002 (50.3%) patients were assigned to VL and 1979 (49.7%) were assigned to DL. The mean age of patients in individual studies ranged from 37 to 69 years, and the proportion of males was 63.7%. Table 1 summarizes the main characteristics of the included studies.

**Table 1:** Baseline characteristics of included studies

First Author, Year^Ref. #^ (Study Acronym or Registry)	Settings	Number of patients	Mean age (y)	Devices	Difficult airway	Intubators’ experience*	RSI	NMBAs
Prekker et al. [23] (DEVICE)	ICU and ED	1417	52	Any VL vs. DL	9.1%	Experienced	NA	Most
Dharanindra et al. [15]	ICU	143	48	King Vision vs. DL	21.7%	Experienced	All	All
Ajith et al. [14]	ED	76	NA	McGrath MAC vs. DL	0%	Inexperienced	DSI	None
Sanguanwit et al. [24]	ED	158	69	GlideScope vs. DL	12.7%	Inexperienced	Most	NA
Dey et al. [25]	ICU	218	47	C-MAC vs. Macintosh	NA	Experienced	NA	Most
Abdelgalel and Mowafy [26]	ICU	120	43	GlideScope vs. Airtraq vs. DL	NA	Experienced	All	All
Gao et al. [27]	ICU	167	69	UEScope vs. DL	9.2%	Unclear	NA	None
Lascarrou et al. [11] (MACMAN)	ICU	371	68	McGrath MAC vs. DL	19.4%	Inexperienced	NA	All
Driver et al. [28]	ED	198	52	C-MAC vs. DL	27.3%	Experienced	Most	Most
Goksu et al. [33]	ED	150	37	C-MAC vs. DL	NA	Inexperienced	All	NA
Janz et al. [29] (FELLOW)	ICU	150	59	McGrath MAC (98.6%), GlideScope (1.4%) vs DL	4.0%	Experienced	NA	All
Sulser et al. [30]	ED	150	54	C-MAC vs. DL	NA	Experienced	All	As needed
Yeatts et al. [31]	ED	623	44	GlideScope vs. DL	NA	Inexperienced	All	All
Griesdale et al. [32] (VICI)	ICU	40	65	GlideScope vs. DL	15.0%	Inexperienced	All	All

*Abbreviations:* DL; direct laryngoscopy; ED; emergency department; ICU; intensive care unit; RCT; randomized controlled trial; RSI; rapid sequence intubation; DSI, delayed sequence intubation; VL; videolaryngoscopy. Notes: * Defined in Additional file 1: Supplemental Methods 5

### Efficacy endpoints

**Fig. 2:**
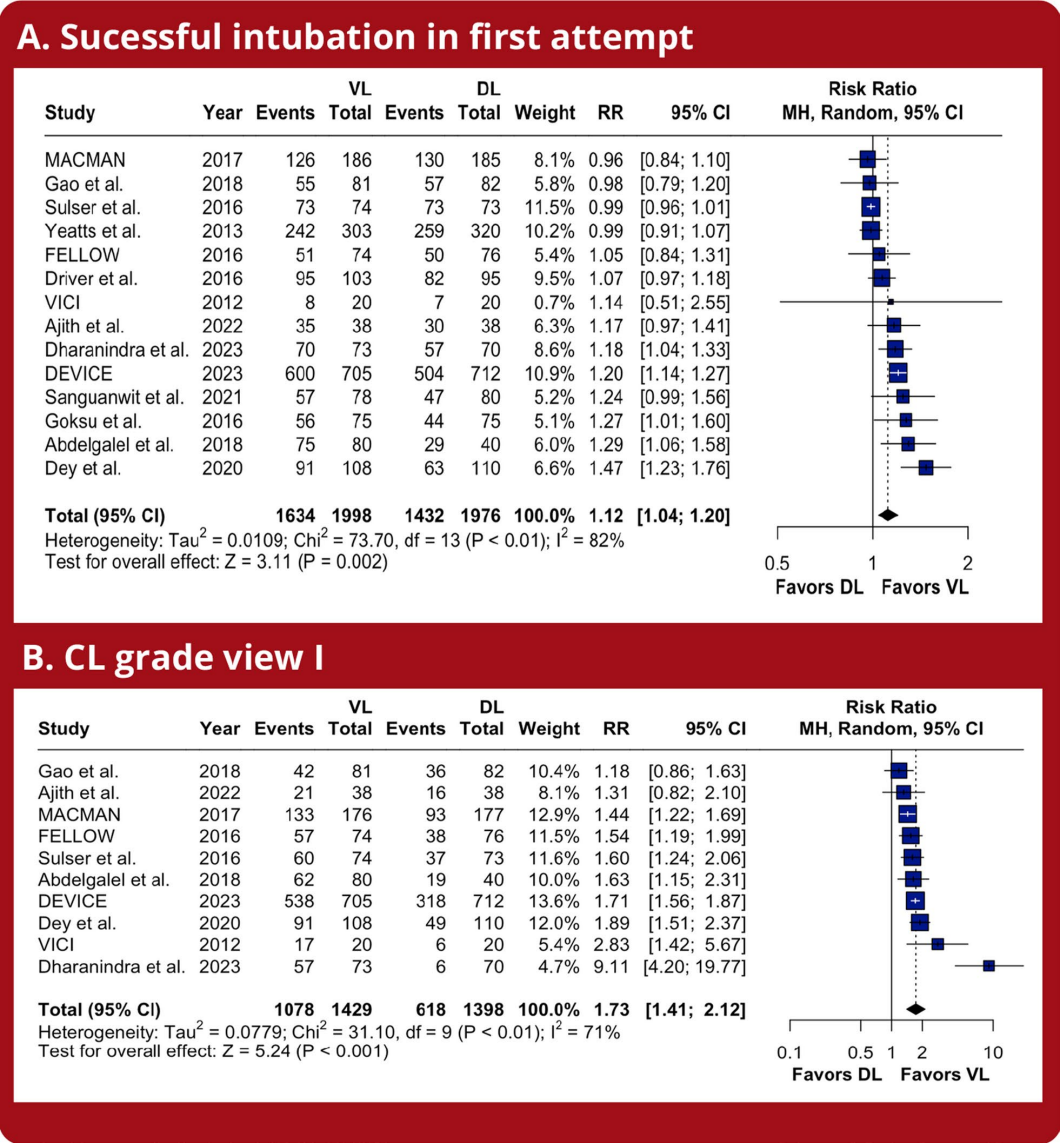
Meta-analysis of the efficacy endpoints in critically ill patients undergoing intubation with VL. *Caption:* Forest plots presenting the risk ratio (RR) and 95% confidence interval (CI) for each strategy on **A** successful intubation on the first attempt and **B** Cormack Lehane (CL) Grade I. *Abbreviations:* CI, confidence interval; MH, Mantel–Haenszel; DL, direct laryngoscope; VL, video laryngoscope; RR, risk ratio; CL, Cormack Lehane

**Fig. 3:**
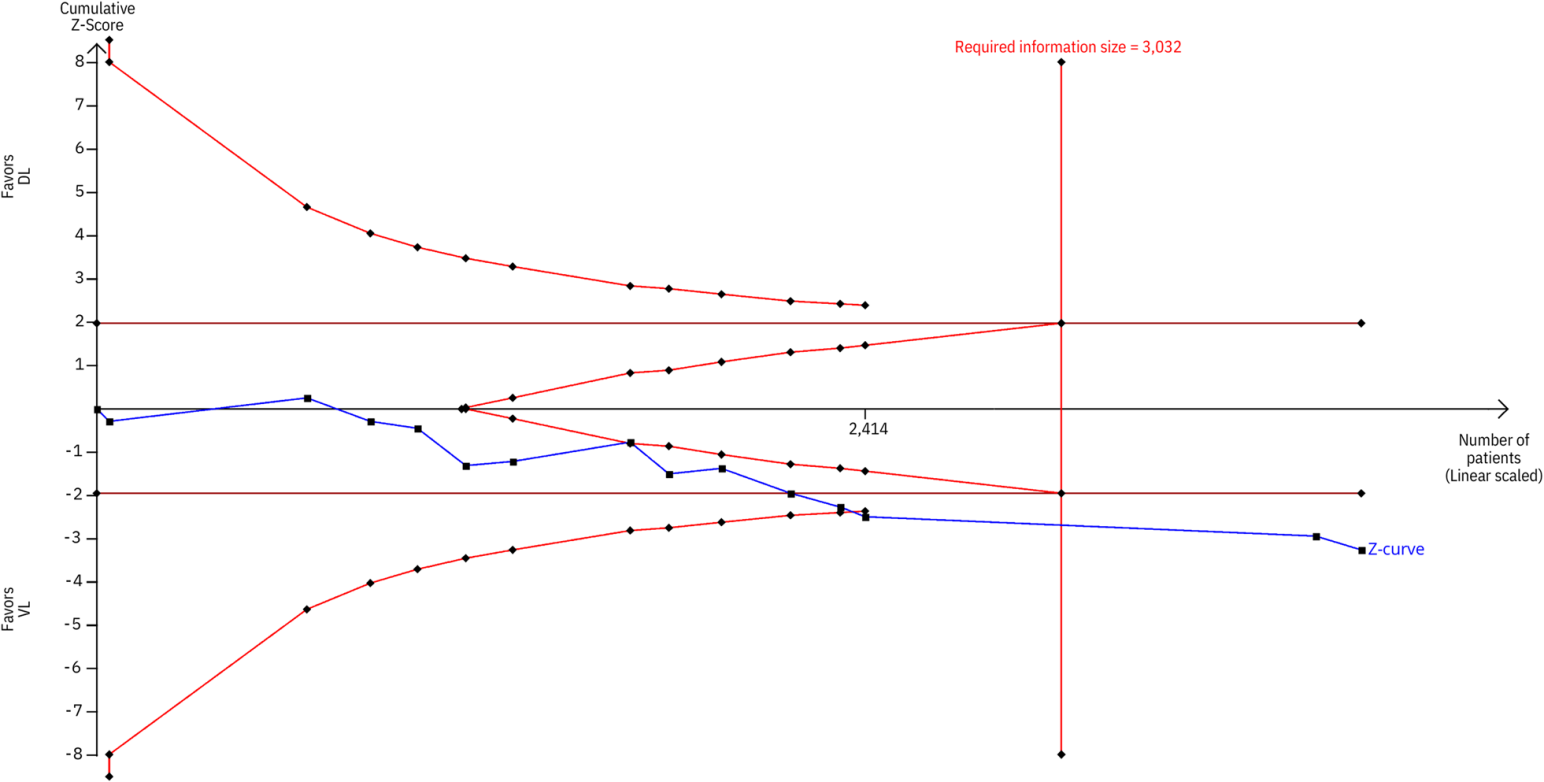
Trial sequential analysis of successful intubation on the first attempt with VL. *Abbreviations:* DL, direct laryngoscope; VL, video laryngoscope

Compared with DL, VL significantly increased the number of successful intubations on the first attempt (RR 1.12; 95% CI 1.04–1.20; *p* < 0.01; *I*^2^ = 82%; Fig. 2A), the proportion of CL grade I (RR 1.73; 95% CI 1.41–2.12; *p* < 0.01; *I*^2^ = 71%; Fig. 2B), and grade I/II (RR 1.12; 95% CI 1.04–1.19; *p* < 0.01; *I *^2^ = 91%; Additional 1: Supplemental Fig. 1). However, no statistically significant difference was observed between the groups in terms of success on the second attempt (RR 1.04; 95% CI 0.94–1.15; *p* = 0.49; *I*^2^ = 83%; Supplemental Fig. 2). Regarding TSA, the cumulative Z-curve crossed the required information size obtained with the 3032 subjects, indicating a low chance of type 1 error for successful intubation on the first attempt (Fig. 3).

### Safety endpoints

**Fig. 4:**
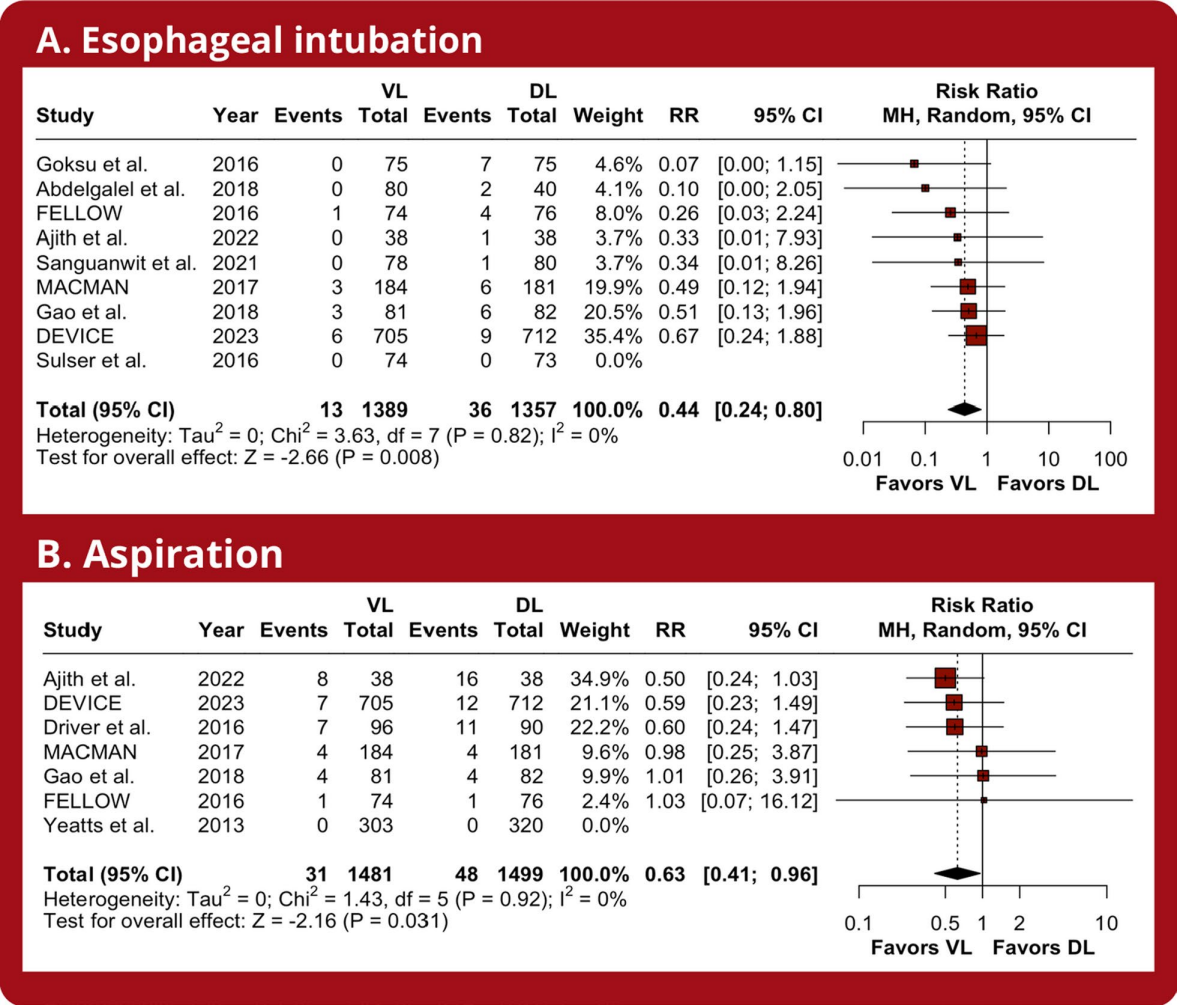
Meta-analysis of safety endpoints in critically ill patients undergoing intubation with VL. *Caption:* Forest plots presenting the risk ratio (RR) and 95% confidence interval (CI) for each strategy on **A** esophageal intubation and **B** aspiration. *Abbreviations:* CI, confidence interval; MH, Mantel–Haenszel; DL, direct laryngoscope; VL, video laryngoscope; RR, risk ratio

VL substantially reduced the number of esophageal intubations (RR 0.44; 95% CI 0.24–0.80; *p* < 0.01; *I*^2^ = 0%; Fig. 4A) and aspirations (RR 0.63; 95% CI 0.41–0.96; *p* = 0.03; *I*^2^ = 0%; Fig. 4B) compared to DL. However, there were similar incidences of dental injury (RR 0.67; 95% CI 0.20–2.24; *p* = 0.51; *I*^2^ = 0%; Additional 1: Supplemental Fig. 3A), cardiac arrest (RR 1.66; 95% CI 0.52–5.30; *p* = 0.39; *I*^2^ = 0%; Additional 1: Supplemental Fig. 3B), all-cause mortality (RR 1.00; 95% CI 0.87–1.16; *p* = 0.95; *I*^2^ = 0%; Additional 1: Supplemental Fig. 3C), and severe hypoxemia (RR 0.98; 95% CI 0.74–1.29; *p* = 0.87; *I*^2^ = 22%; Additional 1: Supplemental Fig. 3D).

### Subgroup and sensitivity analysis

**Fig. 5:**
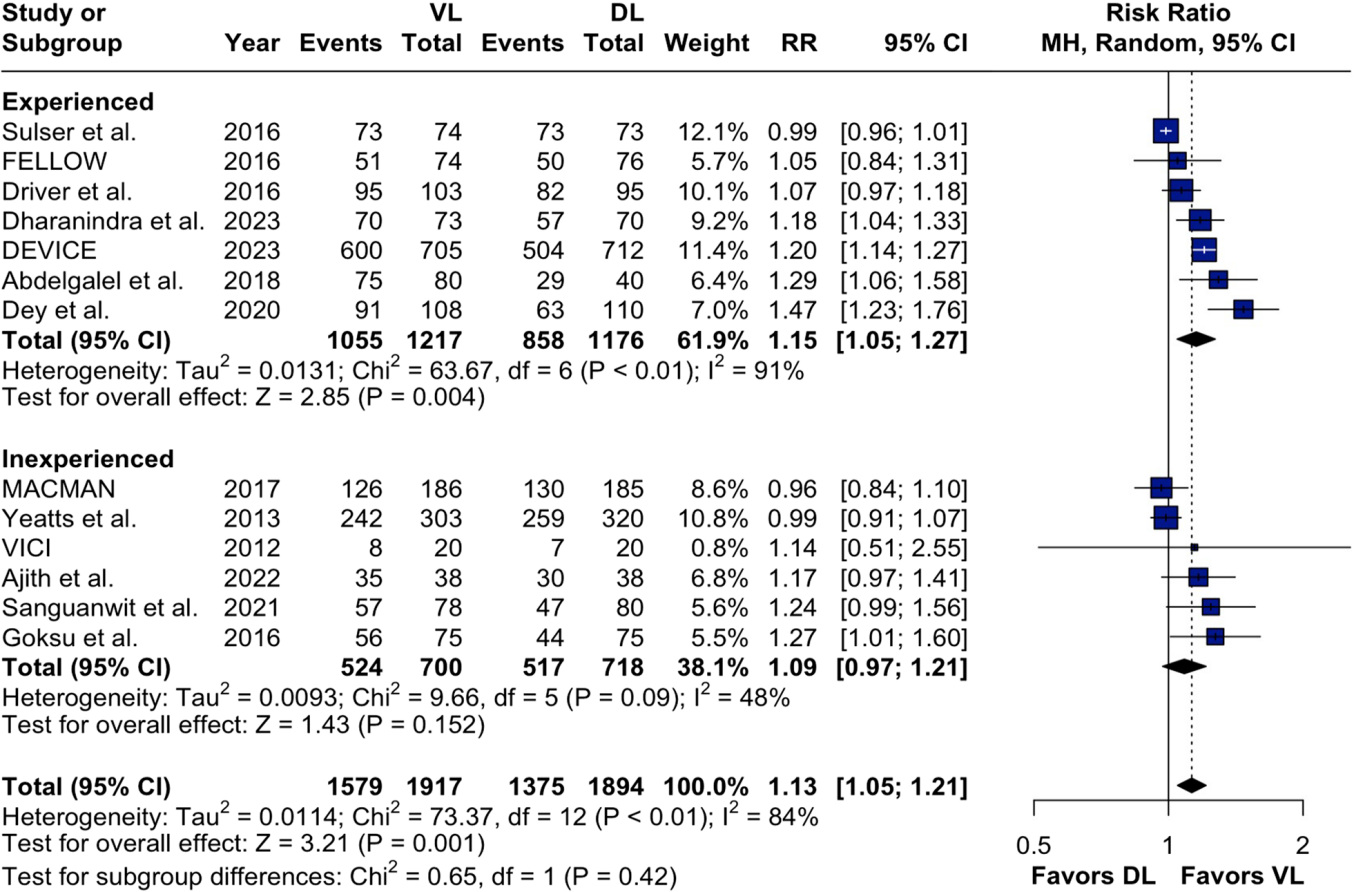
Subgroup analysis of primary outcome by operator's experience in intubating critically ill patients with VL. *Abbreviations:* CI, confidence interval; MH, Mantel–Haenszel; DL, direct laryngoscope; VL, video laryngoscope; RR, risk ratio

There was a significant subgroup interaction among the brands of VL employed (*p* = 0.03; Additional 1: Supplemental Fig. 4A). C-MAC and GlideScope performed similarly, but with McGrath MAC, there was no significant difference between VL and DL (RR 0.99; 95% CI 0.96–1.02; *p* = 0.43; *I*^2^ = 11%). Furthermore, there were no significant subgroup interactions when analyzing subgroups stratified by settings (ICU versus ED) (*p* = 0.48; Additional 1: Supplemental Fig. 4B) or operators’ experience (*p* = 0.42; Fig. 5). In sensitivity analysis changing the threshold from 50 to 100 prior intubations to be considered experienced, there was no significant subgroup interaction (*p* = 0.53; Additional 1: Supplemental Fig. 5).

### Addressing heterogeneity

We conducted a Graphic Display of Heterogeneity (GOSH) analysis to investigate the moderate to high heterogeneity in our findings. Our results were consistent across multiple simulations and remained stable after random exclusion of studies. We identified one study as the main outlier [25]. A comprehensive explanation of statistical protocols used to explore heterogeneity is available in Additional 1: Supplemental Results 1 and Additional 1: Supplemental Figs. 6–8, 11, and 12.

### Risk of bias assessment

Individual RCT appraisal can be found in Additional 1: Supplemental Fig. 9. Regarding the primary outcome, thirteen studies carried high risk of bias due to unblinding of outcome adjudicators due to the nature of intervention, however the DEVICE trial was scored at a low risk of bias due to the presence of an independent observer keeping track of the number of intubation attempts [12]. Moreover, seven studies had some concerns of bias due to the inexistence of protocols [14, 15, 24–27, 33]. Funnel plot and Egger’s test (p = 0.048) suggested publication bias in the primary outcome, as represented in Additional 1: Supplemental Fig. 10.

## Discussion

This meta-analysis of 14 RCTs, encompassing 3981 patients, compared the efficacy and safety of VL in critically ill patients. Our main findings were as follows: (1) VL resulted in higher rates of successful intubations on first attempt; (2) VL led to improved glottic visualization; and (3) VL reduced the incidence of esophageal intubations.

Comprehensive guidelines for managing the intubation of critically ill adults have acknowledged the advantages of VL and recommended its ready availability, considering it the preferred option for all intubations of critically ill patients [34–36]. In contrast, these recommendations were not based on previous meta-analyses of RCTs, in which there was no statistically significant benefit of VL over DL in terms of successful intubation on the first attempt [7].

Notably, the performance of VL could be different between brands owing to various designs and shapes [37, 38]. In our analysis, we included three VL blade design (hyperangulated, standard geometry, or channeled), including six different brands (GlideScope, C-MAC, McGrath MAC, UEScope, KingVision, and Airtraq). Interestingly, in the subgroup analysis comparing different VL brands, we found a potential interaction between the VL manufacturer and treatment effect. The benefit of VL over DL tended to be higher with GlideScope and C-MAC. Future head-to-head comparison studies are warranted for conclusive evidence between VL manufacturers.

Our study showed a substantially lower incidence of esophageal intubation and aspiration during tracheal intubation when utilizing VL. Despite of the substantial increase in the rates of successful intubations on the first attempt, it is noteworthy that this did not lead to significant reduction in all-cause mortality, severe hypoxemia, or cardiac arrest.

Regarding operators’ experience among providers, there were different definitions of experience among studies, which we addressed by classifying them through specific criteria (Additional 1: Supplemental Methods 5). To assess the impact of this important variable, we performed two subgroup analyses, in which there was no statistically significant subgroup interaction with a threshold of mean 50 prior intubations (*p* = 0.42) or 100 prior intubations to be considered experienced (*p* = 0.53); however, limitations must be acknowledged. One study was unclearly defined as per our criteria, limiting the complete evaluation of this analysis [27].

The choice of sedatives and analgesics for induction could also add heterogeneity to our findings. Rapid sequence induction with sedatives and neuromuscular blocking agents has been shown to facilitate tracheal intubation and decrease intubation-related complications in reasonable circumstances [39]. Due to the lack of strict protocols regarding medication in most of the studies included in this review, subgroup analysis based on medications was not feasible.

Although there is a previous meta-analysis on this issue, our study has some advantages. First, we included 7 additional RCTs compared to the previous study [13]. Second, to minimize potential confounders, we excluded quasi-RCT studies. Third, we restricted our inclusion criteria to patients who potentially derive the most benefit from VL (in the ICU and ED settings). Fourth, key findings were revealed: VL led to higher success rate of intubation on the first attempt compared with DL; and VL reduced the incidence of esophageal intubations.

### Study limitations

This meta-analysis has some limitations. First, there was a substantial heterogeneity in the primary outcome. However, we meticulously addressed this heterogeneity by exploring the potential study-level characteristics, as reported in the Additional 1: Supplementary Appendix. Second, our analysis indicated the presence of publication bias concerning the primary outcome. Third, we identified an elevated risk of bias due to the outcome adjudication of the primary outcome, primarily because blinding was impossible due to its inherent nature. Fourth, aspiration relied on operator-reported data, which may be subject to reporting bias. Fifth, only one of included studies reported the presence of secretion as a reason of intubation failure. Therefore, it was not possible to analyze this important variable. Sixth, the subgroup analysis on different VL brands used by individual studies should be interpreted cautiously, as different manufacturers could provide both standard geometry and hyperangulated blades, which impact could not be analyzed. Finally, the absence of patient-level data precluded a more granular assessment of factors potentially related to the relative efficacy of VL vs. DL, such as the operators’ experience and proportion of patients with difficult airways.

## Conclusion

In this meta-analysis of RCTs, in critically ill patients, VL led to a higher number of successful intubations on the first attempt, improved visualization through CL grading, and reduced esophageal intubations compared with DL. Our findings support the routine use of VL in critically ill patients.

### Supplementary Information

**Additional file 1**. Supplementary appendix.

## Data Availability

All data generated or analyzed during this study are included within the published article and its additional files.

## Abbreviations

CI: Confidence interval
CL: Cormack Lehane
DEVICE: Direct versus Video Laryngoscope Trial
DL: Direct laryngoscope
ED: Emergency department
ICU: Intensive care unit
MD: Mean difference
PRISMA: Preferred Reporting Items for Systematic Reviews and Meta-Analysis
RCT: Randomized controlled trial
PROSPERO: International Prospective Register of Systematic Reviews
RR: Risk ratio
TSA: Trial sequential analysis
VL: Video laryngoscope
