# Supplementary material for: Video versus direct laryngoscopy in critically ill patients: an updated systematic review and meta-analysis of randomized controlled trials
Source: Crit Care. 2024 Jan 2;28:1. doi: 10.1186/s13054-023-04727-9 (PMC10759602; doi:10.1186/s13054-023-04727-9)
Supplement: Supplementary file 1 — Additional file 1. Supplementary appendix. [file 13054_2023_4727_MOESM1_ESM.docx]

**SUPPLEMENTARY APPENDIX**

**Video versus Direct Laryngoscopy for Critically Ill Patients: An Updated Systematic Review and Meta-Analysis of Randomized Controlled Trials**

Beatriz Araújo,^1^ André Rivera, ^1^ Suzany Martins,^1^ Renatha Abreu, ^1^ Paula Cassa ^1^, Maicon Silva,^1^ Alice Gallo de Moraes, MD^2^

*^1^Department of Medicine, Nove de Julho University, São Bernardo do Campo, Brazil*

***^2^*** *Division of Pulmonary and Critical Care Medicine, Mayo Clinic, Rochester, MN, USA*

**Table of Contents**

[Supplemental Methods 1. PRISMA 2020 Main Checklist 3](#_Toc149059605)

[Supplemental Methods 2. PRISMA Abstract Checklist 3](#_Toc149059606)

[Supplemental Methods 3. Details of the Search Strategy 4](#_Toc149059607)

[Supplemental Methods 4. Main Exclusion Criteria Used by Study 5](#_Toc149059608)

[Supplemental Methods 5. Operators’ Experience Definition 7](#_Toc149059609)

[Supplemental Methods 6. Study Selection of Device for Intubation on the Second Attempt 10](#_Toc149059610)

[Supplemental Methods 7. Definitions of Outcomes 11](#_Toc149059611)

[Supplemental Methods 8. Addressing Heterogeneity 12](#_Toc149059612)

[Supplemental Results 1. Addressing heterogeneity 13](#_Toc149059613)

[Supplemental Figure 1. Cormack Lehane Laryngeal View Grade 1/2 14](#_Toc149059614)

[Supplemental Figure 2. Successful Intubation on the Second Attempt 15](#_Toc149059615)

[Supplemental Figure 3. Secondary Safety Endpoints 16](#_Toc149059616)

[Supplemental Figure 4. Subgroup Analysis for the Primary Endpoint 17](#_Toc149059617)

[Supplemental Figure 5. Subgroup Analysis Stratified by Operator’s Experience (Using a Threshold of 100 Prior Intubations) 19](#_Toc149059618)

[Supplemental Figure 6. Graphical Display of Study Heterogeneity (GOSH) 20](#_Toc149059619)

[Supplemental Figure 7. Baujat Plot of the Primary Efficacy Endpoint 23](#_Toc149059620)

[Supplemental Figure 8. Leave-one-out Sensitivity Analysis of the Primary Efficacy Endpoint 24](#_Toc149059621)

[Supplemental Figure 9. Risk of Bias 2 of All Included Studies 25](#_Toc149059622)

[Supplemental Figure 10. Funnel Plot and Egger’s Test for the Primary Efficacy Endpoint 26](#_Toc149059623)

[Supplemental Figures 11. Sensitivity Analyses of the Primary Outcome 27](#_Toc149059624)

[Supplemental Figures 12. *Influence.Analysis* and *find.outliers* R Functions for the Primary Outcome 28](#_Toc149059625)

[Supplemental References 29](#_Toc149059626)

# Supplemental Methods 1. PRISMA 2020 Main Checklist

| **Topic** | **No.** | **Item** | **Location where item is reported** |
| --- | --- | --- | --- |
| **TITLE** |  |  |  |
| **Title** | 1 | Identify the report as a systematic review. | Pg. 1 at MS |
| **ABSTRACT** |  |  |  |
| **Abstract** | 2 | See the PRISMA 2020 for Abstracts checklist | NA |
| **INTRODUCTION** |  |  |  |
| **Rationale** | 3 | Describe the rationale for the review in the context of existing knowledge. | Pg. 4 at MS |
| **Objectives** | 4 | Provide an explicit statement of the objective(s) or question(s) the review addresses. | Pg. 4 at MS |
| **METHODS** |  |  |  |
| **Eligibility criteria** | 5 | Specify the inclusion and exclusion criteria for the review and how studies were grouped for the syntheses. | Pg 5-8 at MS |
| **Information sources** | 6 | Specify all databases, registers, websites, organizations, reference lists and other sources searched or consulted to identify studies. Specify the date when each source was last searched or consulted. | Pg. 5 at MS |
| **Search strategy** | 7 | Present the full search strategies for all databases, registers, and websites, including any filters and limits used. | Pg. 4 at sup |
| **Selection process** | 8 | Specify the methods used to decide whether a study met the inclusion criteria of the review, including how many reviewers screened each record and each report retrieved, whether they worked independently, and if applicable, details of automation tools used in the process. | Pg. 5 at MS |
| **Data collection process** | 9 | Specify the methods used to collect data from reports, including how many reviewers collected data from each report, whether they worked independently, any processes for obtaining or confirming data from study investigators, and if applicable, details of automation tools used in the process. | Pg. 6 at MS |
| **Data items** | 10a | List and define all outcomes for which data were sought. Specify whether all results that were compatible with each outcome domain in each study were sought (e.g., for all measures, time points, analyses), and if not, the methods used to decide which results to collect. | Pg. 6,7 at MS |
|  | 10b | List and define all other variables for which data were sought (e.g., participant and intervention characteristics, funding sources). Describe any assumptions made about any missing or unclear information. | Pg. 6 at MS |
| **Study risk of bias assessment** | 11 | Specify the methods used to assess risk of bias in the included studies, including details of the tool(s) used, how many reviewers assessed each study and whether they worked independently, and if applicable, details of automation tools used in the process. | Pg. 7 at MS |
| **Effect measures** | 12 | Specify for each outcome the effect measure(s) (e.g., risk ratio, mean difference) used in the synthesis or presentation of results. | Pg 7-8 at MS |
| **Synthesis methods** | 13a | Describe the processes used to decide which studies were eligible for each synthesis (e.g., tabulating the study intervention characteristics and comparing against the planned groups for each synthesis (item 5)). | Table 1 |
|  | 13b | Describe any methods required to prepare the data for presentation or synthesis, such as handling of missing summary statistics, or data conversions. | Pg. 7,8 at MS |
|  | 13c | Describe any methods used to tabulate or visually display results of individual studies and syntheses. | Pg. 7,8 at MS, Pg. 12 at sup. |
|  | 13d | Describe any methods used to synthesize results and provide a rationale for the choice(s). If meta-analysis was performed, describe the model(s), method(s) to identify the presence and extent of statistical heterogeneity, and software package(s) used. | Pg. 7,8 at MS, Pg. 12 at sup. |
|  | 13e | Describe any methods used to explore possible causes of heterogeneity among study results (e.g., subgroup analysis, meta-regression). | Pg. 7 at MS |
|  | 13f | Describe any sensitivity analyses conducted to assess robustness of the synthesized results. | Pg. 7 at MS, Pg. 10,11 at sup |
| **Reporting bias assessment** | 14 | Describe any methods used to assess risk of bias due to missing results in a synthesis (arising from reporting biases). | Pg. 7 at MS |
| **Certainty assessment** | 15 | Describe any methods used to assess certainty (or confidence) in the body of evidence for an outcome. | NA |
| **RESULTS** |  |  |  |
| **Study selection** | 16a | Describe the results of the search and selection process, from the number of records identified in the search to the number of studies included in the review, ideally using a flow diagram. | Figure 1 |
|  | 16b | Cite studies that might appear to meet the inclusion criteria, but which were excluded, and explain why they were excluded. | NA |
| **Study characteristics** | 17 | Cite each included study and present its characteristics. | Table 1, Pg. 8 at MS |
| **Risk of bias in studies** | 18 | Present assessments of risk of bias for each included study. | Pg. 10 at MS, Pg. 25 at sup. |
| **Results of individual studies** | 19 | For all outcomes, present, for each study: (a) summary statistics for each group (where appropriate) and (b) an effect estimates and its precision (e.g., confidence/credible interval), ideally using structured tables or plots. | Fig. 2,4, and 5 at MS, Pg. 14-19 at sup. |
| **Results of syntheses** | 20a | For each synthesis, briefly summarize the characteristics and risk of bias among contributing studies. | Pg. 25 at sup. |
|  | 20b | Present results of all statistical syntheses conducted. If meta-analysis was done, present for each the summary estimate and its precision (e.g., confidence/credible interval) and measures of statistical heterogeneity. If comparing groups, describe the direction of the effect. | Pg 8,9 at MS |
|  | 20c | Present results of all investigations of possible causes of heterogeneity among study results. | Pg. 9,10 at MS, Pg. 13 at sup. |
|  | 20d | Present results of all sensitivity analyses conducted to assess the robustness of the synthesized results. | Pg. 20-24, 27 at sup. |
| **Reporting biases** | 21 | Present assessments of risk of bias due to missing results (arising from reporting biases) for each synthesis assessed. | Pg. 25,26 at sup. |
| **Certainty of evidence** | 22 | Present assessments of certainty (or confidence) in the body of evidence for each outcome assessed. | NA |
| **DISCUSSION** |  |  |  |
| **Discussion** | 23a | Provide a general interpretation of the results in the context of other evidence. | Pg. 10-12 at MS |
|  | 23b | Discuss any limitations of the evidence included in the review. | Pg. 12-13 at MS |
|  | 23c | Discuss any limitations of the review processes used. | Pg. 12-13 at MS |
|  | 23d | Discuss implications of the results for practice, policy, and future research. | Pg. 11, 12 at MS |
| **OTHER INFORMATION** |  |  |  |
| **Registration and protocol** | 24a | Provide registration information for the review, including register name and registration number, or state that the review was not registered. | PROSPERO; CRD42023439685 |
|  | 24b | Indicate where the review protocol can be accessed, or state that a protocol was not prepared. | <https://www.crd.york.ac.uk/prospero/display_record.php?ID=CRD42023439685> |
|  | 24c | Describe and explain any amendments to information provided at registration or in the protocol. | NA |
| **Support** | 25 | Describe sources of financial or non-financial support for the review, and the role of the funders or sponsors in the review. | None |
| **Competing interests** | 26 | Declare any competing interests of review authors. | Pg. 1 |
| **Availability of data, code and other materials** | 27 | Report which of the following are publicly available and where they can be found template data collection forms; data extracted from included studies; data used for all analyses; analytic code; any other materials used in the review. | NA |

^Abbreviations: MS, manuscript; sup., supplement.^

# Supplemental Methods 2. PRISMA Abstract Checklist

| **Topic** | **No.** | **Item** | **Reported?** |
| --- | --- | --- | --- |
| **TITLE** |  |  |  |
| **Title** | 1 | Identify the report as a systematic review. | Yes |
| **BACKGROUND** |  |  |  |
| **Objectives** | 2 | Provide an explicit statement of the main objective(s) or question(s) the review addresses. | Yes |
| **METHODS** |  |  |  |
| **Eligibility criteria** | 3 | Specify the inclusion and exclusion criteria for the review. | Yes |
| **Information sources** | 4 | Specify the information sources (e.g., databases, registers) used to identify studies and the date when each was last searched. | Yes |
| **Risk of bias** | 5 | Specify the methods used to assess risk of bias in the included studies. | No |
| **Synthesis of results** | 6 | Specify the methods used to present and synthesize results. | Yes |
| **RESULTS** |  |  |  |
| **Included studies** | 7 | Give the total number of included studies and participants and summarize relevant characteristics of studies. | Yes |
| **Synthesis of results** | 8 | Present results for main outcomes, preferably indicating the number of included studies and participants for each. If meta-analysis was done, report the summary estimate and confidence/credible interval. If comparing groups, indicate the direction of the effect (i.e., which group is favored). | Yes |
| **DISCUSSION** |  |  |  |
| **Limitations of evidence** | 9 | Provide a brief summary of the limitations of the evidence included in the review (e.g., study risk of bias, inconsistency and imprecision). | No |
| **Interpretation** | 10 | Provide a general interpretation of the results and important implications. | No |
| **OTHER** |  |  |  |
| **Funding** | 11 | Specify the primary source of funding for the review. | No |
| **Registration** | 12 | Provide the register name and registration number. | Yes |

# Supplemental Methods 3. Details of the Search Strategy

| **Search Strategy for each database** | |
| --- | --- |
| Pubmed | video* AND (laryngoscop*[Title/Abstract] OR intubation[Title/Abstract] OR videolaryngoscop*[Title/Abstract] OR "airway scope"[Title/Abstract] OR "video laryngoscopic"[Title/Abstract] OR "video laryngoscope"[Title/Abstract]) AND (randomized[Title/Abstract] OR randomised[Title/Abstract] OR random[Title/Abstract] OR randomly[Title/Abstract] OR RCT[Title/Abstract]) |
| EMBASE | ('video' OR 'videolaryngoscope' OR 'videolaryngoscopic' OR 'video laryngoscope' OR 'video laryngoscope' OR 'video laryngoscopic') AND ('laryngoscope' OR 'intubation' OR 'airway scope') AND ('randomized' OR 'randomised' OR 'random' OR 'randomly' OR 'RCT') |
| Cochrane Library | (("video" OR "videolaryngoscope" OR "videolaryngoscopic" OR "video laryngoscope" OR "video laryngoscopic") AND ("laryngoscope" OR "intubation" OR "airway scope")) AND ("randomized" OR "randomised" OR "random" OR "randomly" OR "RCT") |

# Supplemental Methods 4. Main Exclusion Criteria Used by Study

| **Study and year** | **Main exclusion criteria** |
| --- | --- |
| Prekker, 2023  (DEVICE) | “Patient is known to be less than 18 years old, pregnant or patient is known to be a prisoner. The immediate need for tracheal intubation precludes safe performance of study procedures. Operator has determined that use of a video laryngoscope or use of a direct laryngoscope is required or contraindicated for the optimal care of the patient.” |
| Dharanindra, 2023 | “Patients with upper airway deformities and patients with a known history of subglottic stenosis.” |
| Ajith, 2022 | “Pediatric and crash intubations, extensive airway or maxillo-facial injuries, cervical spine trauma and pregnant patients.” |
| Sanguanwit, 2021 | “Patients who had no signs of undergoing resuscitation and patients who were at the end-of-life care.” |
| Dey, 2020 | “Patients of age less than 18 years, pregnant and lactating women, patients with facial trauma including burn injury, suspected or confirmed cervical spine injury, lack of time for randomization and inclusion due to ongoing resuscitative efforts, unable to obtain informed consent.” |
| Abdelgalel, 2018 | “Patients required endotracheal intubation due to cardiac arrest, severe oxygen desaturation (Spo2 < 80%) and patients with diagnosed or predicted cervical spine injury.” |
| Gao, 2018 | “Contraindications to endotracheal intubation (e.g., unstable spinal lesion). Age younger than 18 years. Currently pregnant or breast feeding.” |
| Lascarrou, 2017  (MACMAN) | “Contraindications to orotracheal intubation (eg, unstable spinal lesion), insufficient time to include and randomize the patient (eg, because of cardiac arrest), age younger than 18 years, currently pregnant or breastfeeding, correctional facility inmate, under guardianship, without health insurance, refusal by patient or next of kin, and previous enrollment in an RCT with intubation as the primary endpoint (including previous inclusion in the present trial).” |
| Driver, 2016 | “Pregnant or a prisoner or if the treating physician planned an approach other than DL on the first intubation attempt.” |
| Goksu, 2016 | “Penetrating trauma, age under 16 and intubated before ED arrival were excluded from the study.” |
| Janz, 2016  (FELLOW) | NA |
| Sulser, 2016 | “Sustained maxillo-facial trauma, immobilised cervical spine, known difficult airway or ongoing cardiopulmonary resuscitation.” |
| Yeatts, 2013 | “Minors were excluded from the study, as were patients with suspected laryngeal trauma or extensive maxillofacial injury who required an immediate surgical airway and patients with known or strongly suspected spinal cord injury for whom awake flexible fiber-optic intubation was indicated. The study also excluded patients in cardiac arrest on arrival as well as those who died in the TRU.” |
| Griesdale, 2012  (VICI) | “Patients were excluded if they required immediate endotracheal intubation (within five minutes) as anticipated by the ICU team, a spontaneous breathing endotracheal intubation technique, or cervical spine precautions. Additional exclusion criteria included a history of (or anticipated) difficult intubation; previous cardiac arrest or cardiopulmonary instability (oxygen saturation [SaO2] \ 90% or systolic blood pressure [SBP]\80 mmHg despite oxygen or fluid and vasopressor therapy); prior clinical deterioration requiring immediate tracheal intubation while awaiting randomization; or deemed inappropriate for enrolment by the attending physician” |

# Supplemental Methods 5. Operators’ Experience Definition

**5A.** Main definition of experience

| **Experienced** | **Non-experienced** |
| --- | --- |
| 1. According to judgement of study authors **OR** 2. Mean/median number of intubations ≥ 50 **OR** 3. All operators being anesthesiologists, emergency medicine, or critical care medicine attending physicians. | 1. Mean or median number of intubations < 50 **OR** 2. Most of intubation performed by medical students 3. All intubations being performed by residents or critical care fellows. |

| **Study and year** | **Experience definition** | **Classification** |
| --- | --- | --- |
| Prekker, 2023  (DEVICE) | “In total, 91.5% of the intubations were performed by an emergency medicine resident or a critical care fellow. Operators had performed a median of **50 previous tracheal intubations (interquartile range, 25 to 92**).” | Experienced |
| Dharanindra, 2023 | “All the intubations in the Macintosh DL group and 98.5% in the KVVL group were performed by the primary author, who was a critical care resident and **qualified anesthetist experienced** in airway management with both DL and VL.” | Experienced |
| Ajith, 2022 | “Intubation was performed by **post-graduate anaesthesia trainee** with at least one year of experience.” | Inexperienced |
| Sanguanwit, 2021 | “First, second, and third-year **emergency residents** or **emergency attending** staff were defined as intubation experiences. **Sixth-year medical students** (under the supervision of emergency residents and emergency attending staff) were defined as not having expertise in intubation.” 75.3% of operators were considered non-experienced. | Inexperienced |
| Dey, 2020 | “Intubating anesthetists (laryngoscopist) had experience of **minimum fifty videolaryngoscopies** using C-MAC VL. Laryngoscopists were categorized (based on exposure) into **junior** (up to three years), **senior** (3–8 years) and **consultant** (more than 8 years) based on their years of **anaesthesia experience**.” | Experienced |
| Abdelgael, 2018 | “Intubation was done by **ICU physician** with more than 3 years of experience in anesthesia and intensive care and performed more than 30 intubations with each of Airtraq and Glidescope.” | Experienced |
| Gao, 2018 | “All the physicians involved had either **worked at ICUs for at least 5 years** or worked at ICUs for at least 1 year after receiving at least 2 months of anesthesiology training.” | Unclear |
| Lascarrou, 2017 (MACMAN) | “An expert was defined as a physician who had either **worked at ICUs for at least 5 years** or worked at ICUs for at least 1 year after receiving **at least 2 years of anesthesiology** training. Physicians who did not meet these criteria were classified as nonexperts.” | Inexperienced |
| Driver, 2016 | “Senior residents (postgraduate year 3 [PGY-3] or higher) performed the majority of tracheal intubations in our institution” | Experienced |
| Goksu, 2016 | Emergency medicine attending physicians performed 11 (7.3%) tracheal intubations.”. Table 1 indicates that 8% of operators are attending physicians. | Inexperienced |
| Janz, 2016  (FELLOW) | Total previous intubations for each group were [median (IQR)]: 68 (52-69) and 56 (47-69) | Experienced |
| Sulser, 2016 | “All intubations were performed by **one of three very experienced anaesthesia consultants**.” | Experienced |
| Yeatts, 2013 | “Emergency medicine or anesthesiology **residents** with a minimum of 1 year of previous intubation experience performed the majority of the procedures under the direct supervision of an attending trauma anesthesiologist.” | Inexperienced |
| Griesdale, 2012  (VICI) | “Both groups included **inexperienced operators**. The primary operators were predominantly **internal medicine residents** (n = 24, 60%) in their first three years of postgraduate training (PGY-1, n = 11, 28%; PGY-2, n =14, 35%; PGY-3, n = 11, 28%). Two medical students acted as primary operators, both in the VL group.” | Inexperienced |

**5B.** Sensitivity analysis changing the threshold to 100 prior intubations

| **Experienced** | **Non-experienced** |
| --- | --- |
| 1. According to judgement of study authors **OR** 2. Mean/median number of intubations ≥ 100 **OR** 3. All operators being anesthesiologists, emergency medicine, or critical care medicine attending physicians. | 1. Mean or median number of intubations < 100 **OR** 2. Most of intubation performed by medical students 3. All intubations being performed by residents or critical care fellows. |

| **Study and year** | **Experience definition** | **Classification** |
| --- | --- | --- |
| Prekker, 2023  (DEVICE) | “In total, 91.5% of the intubations were performed by an emergency medicine resident or a critical care fellow. Operators had performed a median of **50 previous tracheal intubations (interquartile range, 25 to 92**).” | Inexperienced |
| Dharanindra, 2023 | “All the intubations in the Macintosh DL group and 98.5% in the KVVL group were performed by the primary author, who was a critical care resident and **qualified anesthetist experienced** in airway management with both DL and VL.” | Experienced |
| Ajith, 2022 | “Intubation was performed by **post-graduate anaesthesia trainee** with at least one year of experience.” | Inexperienced |
| Sanguanwit, 2021 | “First, second, and third-year **emergency residents** or **emergency attending** staff were defined as intubation experiences. **Sixth-year medical students** (under the supervision of emergency residents and emergency attending staff) were defined as not having expertise in intubation.” 75.3% of operators were considered non-experienced. | Inexperienced |
| Dey, 2020 | “Intubating anesthetists (laryngoscopist) had experience of **minimum fifty videolaryngoscopies** using C-MAC VL. Laryngoscopists were categorized (based on exposure) into **junior** (up to three years), **senior** (3–8 years) and **consultant** (more than 8 years) based on their years of **anaesthesia experience**.” | Experienced |
| Abdelgael, 2018 | “Intubation was done by **ICU physician** with more than 3 years of experience in anesthesia and intensive care and performed more than 30 intubations with each of Airtraq and Glidescope.” | Experienced |
| Gao, 2018 | “All the physicians involved had either **worked at ICUs for at least 5 years** or worked at ICUs for at least 1 year after receiving at least 2 months of anesthesiology training.” | Unclear |
| Lascarrou, 2017 (MACMAN) | “An expert was defined as a physician who had either **worked at ICUs for at least 5 years** or worked at ICUs for at least 1 year after receiving **at least 2 years of anesthesiology** training. Physicians who did not meet these criteria were classified as nonexperts.” | Inexperienced |
| Driver, 2016 | “**Senior residents** (postgraduate year 3 [PGY-3] or higher) performed the majority of tracheal intubations in our institution” | Experienced |
| Goksu, 2016 | Emergency medicine **attending physicians** performed 11 (7.3%) tracheal intubations.”. Table 1 indicates that 8% of operators are attending physicians. | Inexperienced |
| Janz, 2016  (FELLOW) | Total previous intubations for each group were [median (IQR)]: 68 (52-69) and 56 (47-69) | Inexperienced |
| Sulser, 2016 | “All intubations were performed by **one of three very experienced anaesthesia consultants**.” | Experienced |
| Yeatts, 2013 | “Emergency medicine or anesthesiology **residents** with a minimum of 1 year of previous intubation experience performed the majority of the procedures under the direct supervision of an attending trauma anesthesiologist.” | Inexperienced |
| Griesdale, 2012  (VICI) | “Both groups included **inexperienced operators**. The primary operators were predominantly **internal medicine residents** (n = 24, 60%) in their first three years of postgraduate training (PGY-1, n = 11, 28%; PGY-2, n =14, 35%; PGY-3, n = 11, 28%). Two medical students acted as primary operators, both in the VL group.” | Inexperienced |

# Supplemental Methods 6. Study Selection of Device for Intubation on the Second Attempt

| **Study and year** | **Second attempt definition** |
| --- | --- |
| Lascarrou, 2017 (MACMAN) | “If the first-pass intubation attempt failed, the individual performing intubation chose between repeat laryngoscopy and **an alternative intubation technique in accordance with French guidelines**.” |
| Dharanindra, 2023 | “All India Difficult Airway Association guidelines for tracheal intubation in the ICU were followed when intubation **required more than two attempts**. The difficult airway trolley was stocked as per the AIDAA recommendations.” |
| Sanguanwit, 2021 | Before two unsuccessful attempts, they consulted an anesthesiologist or other procedure. |
| Dey, 2020 | “Laryngoscopies were performed using the method as perrandom allocation, **after three failed attempts of intubations alternative techniques were used and subsequently they were not included in the analysis.**” |
| Abdelgalel, 2018 | “Failed endotracheal intubation is defined as failure to intubate the patient after 3 attempts using the same laryngoscope.” |
| Goksu, 2016 | NA |
| Griesdale, 2012  (VICI) | “The number of intubation attempts was the same for both groups. Multiple endotracheal intubation attempts were required in 12 (60%) patients in the VL group and 13 (65%) patients in the DL group**. In the VL group, five of 12 (42%) first attempts with VL failed and resulted in the use of DL for subsequent attempts.** **In contrast, only one of 13 (5%) first attempts failed with DL and resulted in the use of VL for subsequent attempts (P = 0.03**). The supervisor took over in eight of 12 (67%) failed first attempts with DL (with data missing from one patient) compared with four of the 12 (33%) in the VL group (P = 0.22).” |

# Supplemental Methods 7. Definitions of Outcomes

| **Outcome** | **Definition** |
| --- | --- |
| Successful intubation on the first attempt | The proportion of successful placement of a tracheal tube in the trachea during first insertion of a laryngoscope into oral cavity without removing the device from the mouth. Bougies and stylets could be used. |
| Aspiration | Emesis during intubation, witnessed oral content into trachea, or aspiration pneumonia was identified. |
| Severe hypoxemia | Oxygen saturation < 80% |

# Supplemental Methods 8. Addressing Heterogeneity

To ensure the reliability and robustness of our findings, we conducted a thorough analysis to identify potential outliers and influential studies for the primary outcome. This included the use of a Graphic Display of Heterogeneity (GOSH) analysis, in which we performed multiple random simulations and exclusions. The GOSH plot was generated, and three unsupervised machine learning (ML) algorithms were applied to detect clusters in the data, namely the k-means algorithm, density-based spatial clustering of applications with noise (DBSCAN), and Gaussian mixture models.

We created a Baujat plot and performed a leave-one-out sensitivity analysis to investigate potential outliers for the primary efficacy endpoint. The leave-one-out sensitivity analysis removed one study at a time to ensure that our results were not reliant on a single study. These methods helped to identify any potential outliers.

It is worth noting that the MH default continuity correction of 0.5 (default behavior in metabin function [R meta package]) is only necessary when one specific cell is zero in all included studies in the meta-analysis. However, its use in other scenarios has been discouraged by Efthimiou et al. [7], as it can lead to biased results. Therefore, we only applied the continuity correction in the specific situation mentioned above, and when it was not fitted, we used the exact MH method without continuity correction.

# Supplemental Results 1. Addressing Heterogeneity

Hence, due to the significant heterogeneity of the results, we performed Graphical Display of Study Heterogeneity (GOSH) analyses for the primary endpoint. The GOSH plot illustrates the effect size plotted against the *I^2^* for all possible combinations of studies. The 16,383 possible subsets of meta-analysis (2^k^ – 1 possible combinations) for successful intubations at first attempt are presented as a GOSH plot in Supplemental Figure 6A. By analyzing the pattern in our data, we find that most values are concentrated in a cluster with high heterogeneity and a symmetrical distribution along the overall estimate axis. The distribution of *I^2^* is unimodal, with clusters following a high *I^2^*.

To find out which studies cause this shape, we applied three unsupervised machine learning (ML) algorithms, detailed in methods, to detect clusters in the GOSH plot data (Supplemental Figure 6B-6D*)*. Ultimately, one potential outlier was identified similarly by the three unsupervised ML algorithms. The corresponding subset, including this potential outlier, is demonstrated in Supplemental Figure 6F. In summary, the GOSH analysis showed that heterogeneity did not change when Dey et al., 2020 trial was excluded from the analysis. The overall effect did not change significantly before and after excluding random studies in the GOSH plot. However, results also show that the results are stable on multiple simulations, despite significant heterogeneity.

We further explored each study`s influence by performing a Baujat plot leave-one-out sensitivity analysis (Supplemental Figure 7) and plotting the leave-one-out sensitivity analysis (Supplementary Figure 8). The analyses showed that most of the heterogeneity was carried out by Dey et al., 2020 trial, as already identified by GOSH analysis. By inspecting the leave-one-out sensitivity analysis, when the Dey et al., 2020 trial is omitted, the pooled effect estimates (RR) varied from 1.12 to 1.09, accompanied by a slight decrease of heterogeneity (*I^2^*) from 82% to 80%.

# Supplemental Figure 1. Cormack Lehane Laryngeal View Grade 1/2


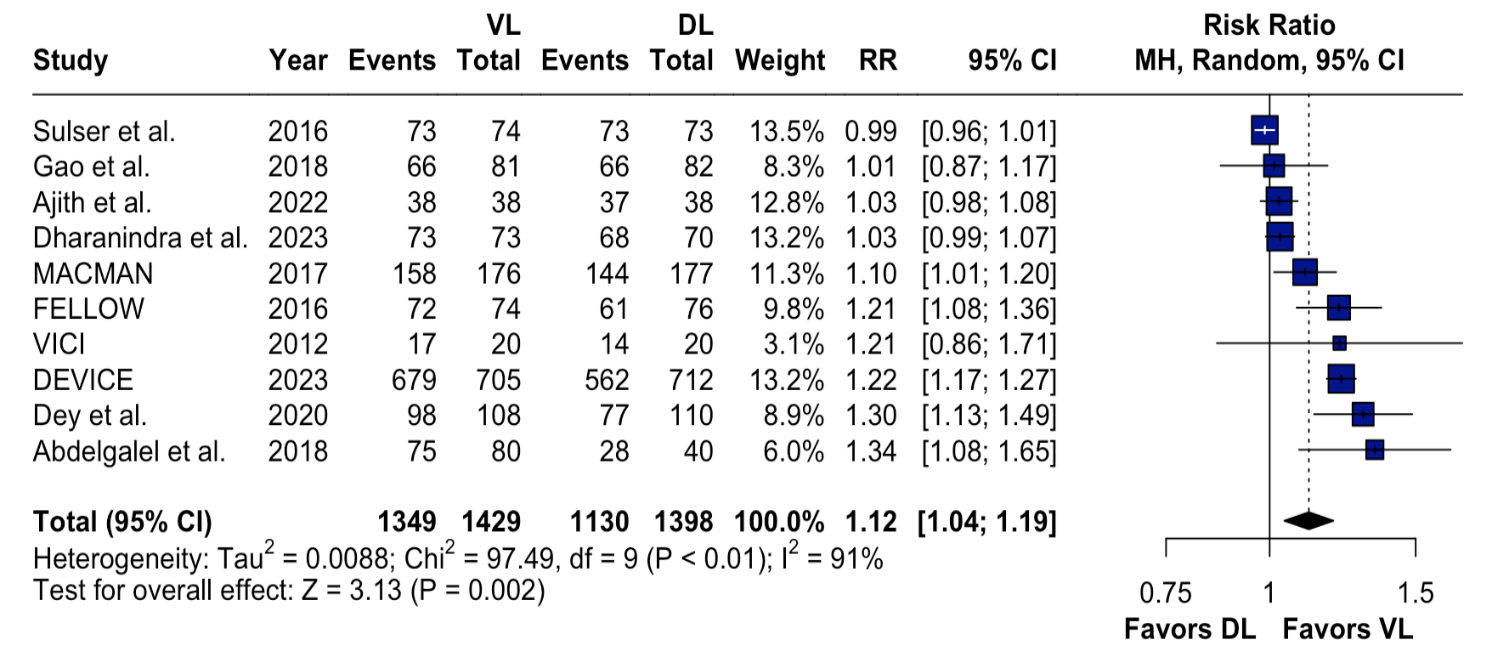


# Supplemental Figure 2. Successful Intubation on the Second Attempt


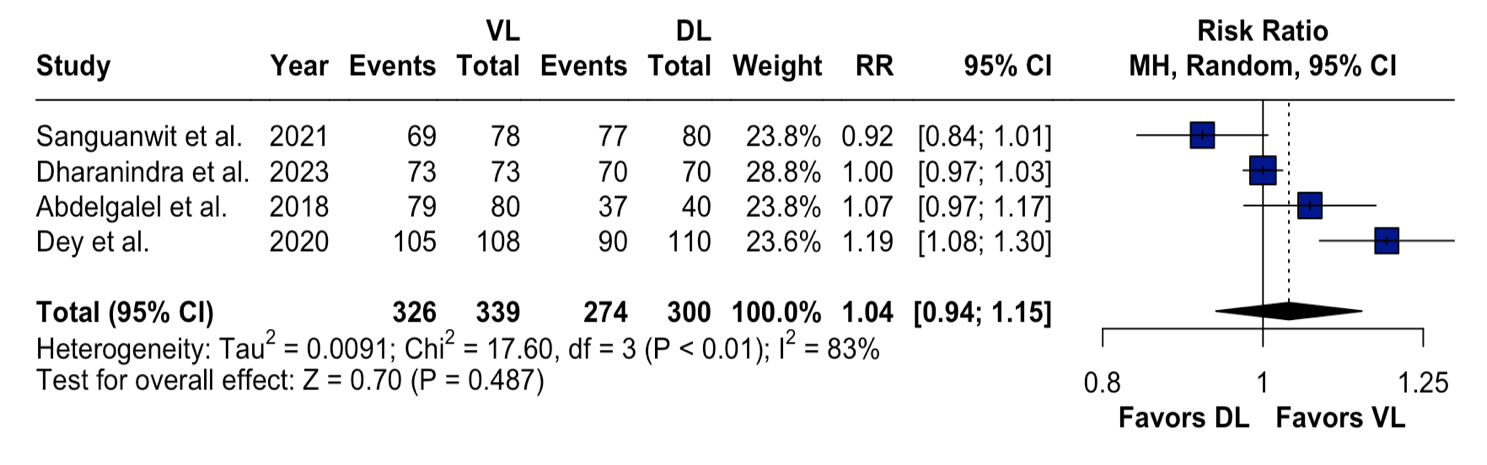


# Supplemental Figure 3. Secondary Safety Endpoints

**Figure 3A.** Dental injury

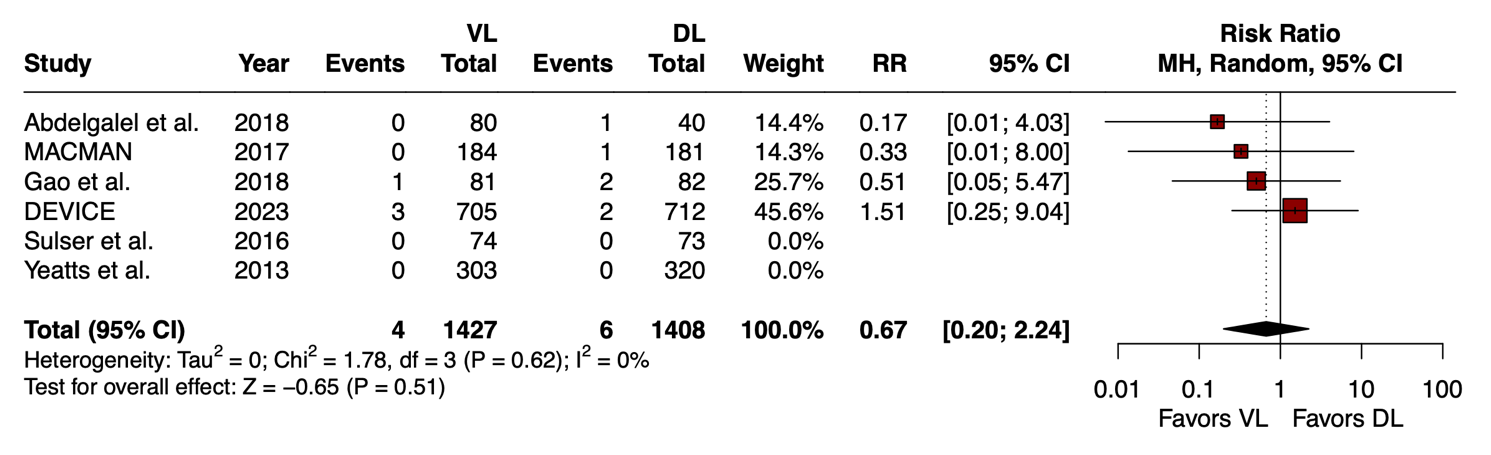

**Figure 3B.** Cardiac arrest

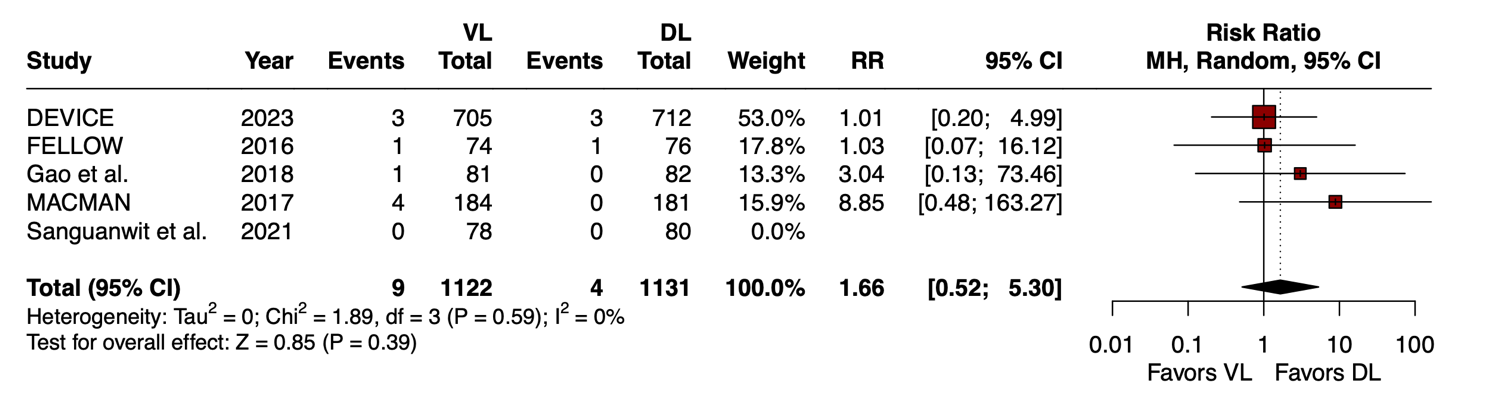

**Figure 3C.** All-cause mortality

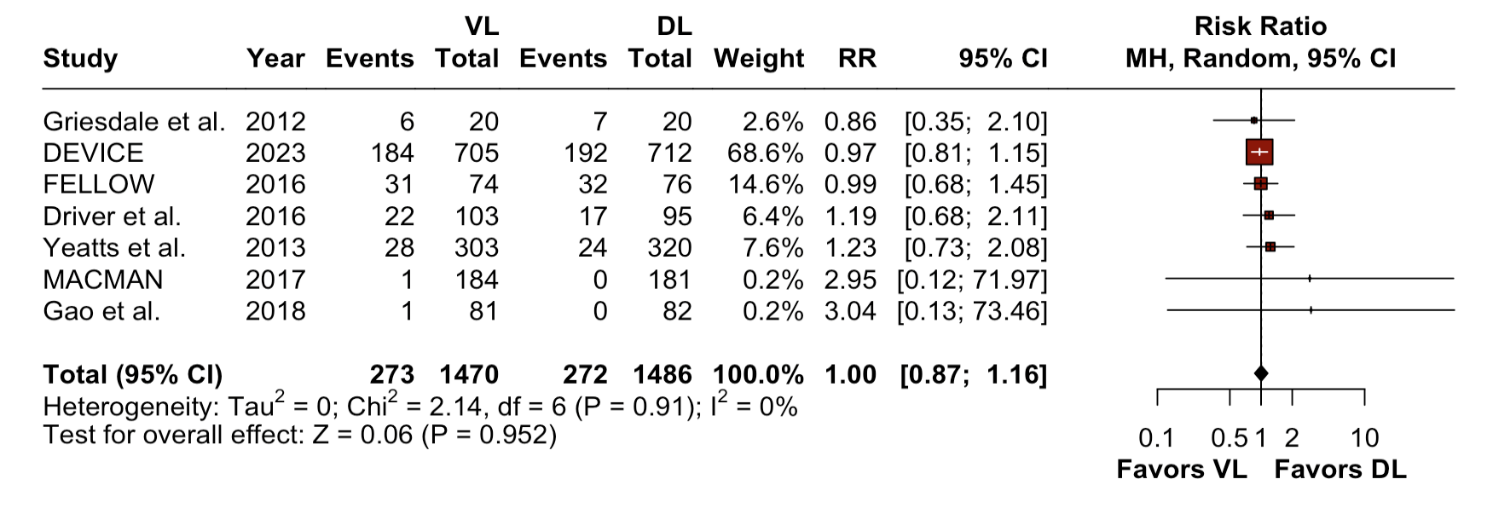

**Figure 3D.** Severe hypoxemia

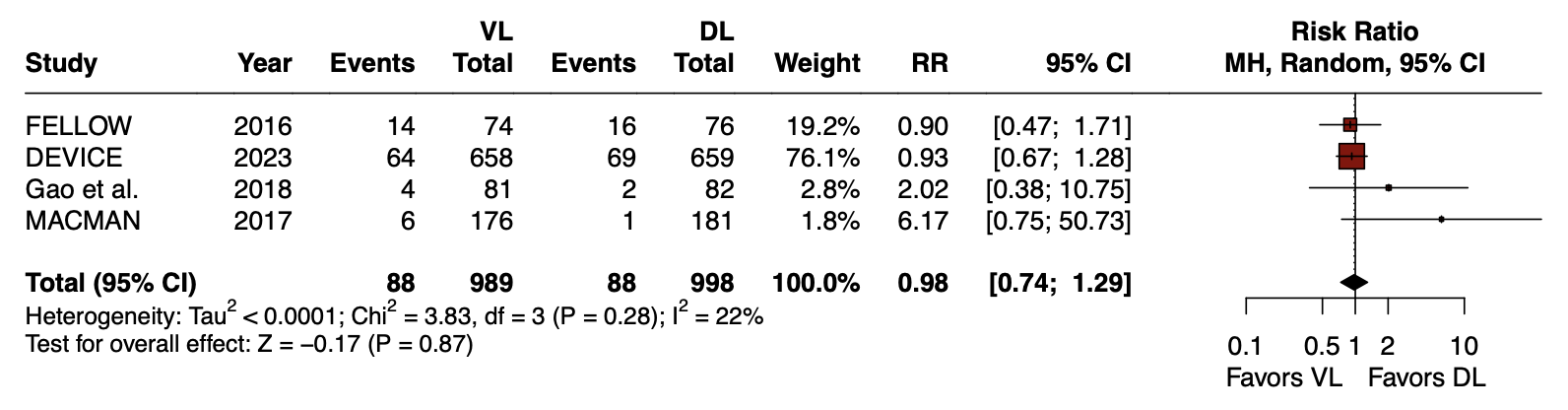


# Supplemental Figure 4. Subgroup Analysis for the Primary Endpoint

**Figure 4A.** Subgroup analysis stratified by VL brands

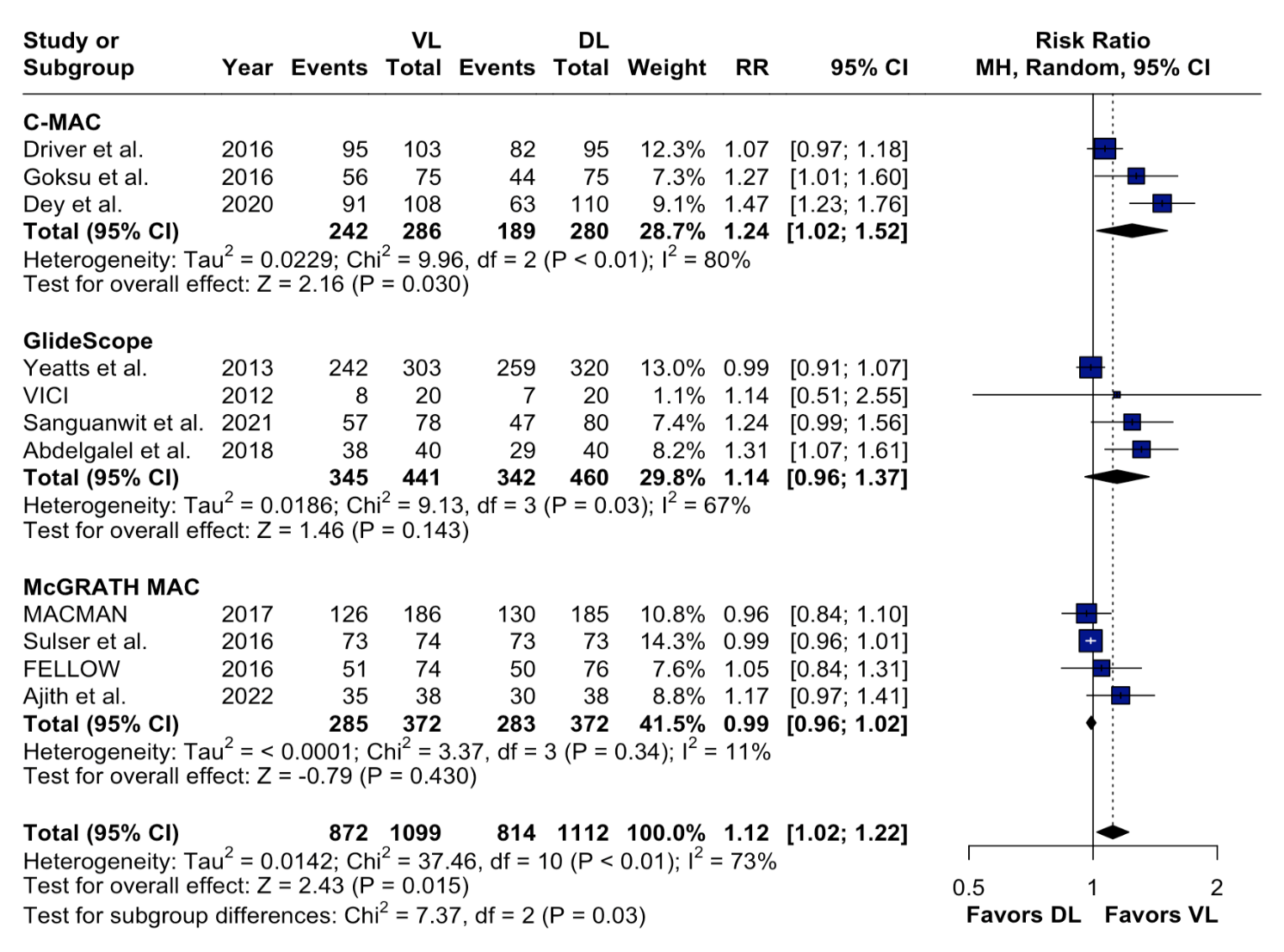


**Figure 4B.** Subgroup analysis stratified by setting (emergency department vs. intensive care unit)

**
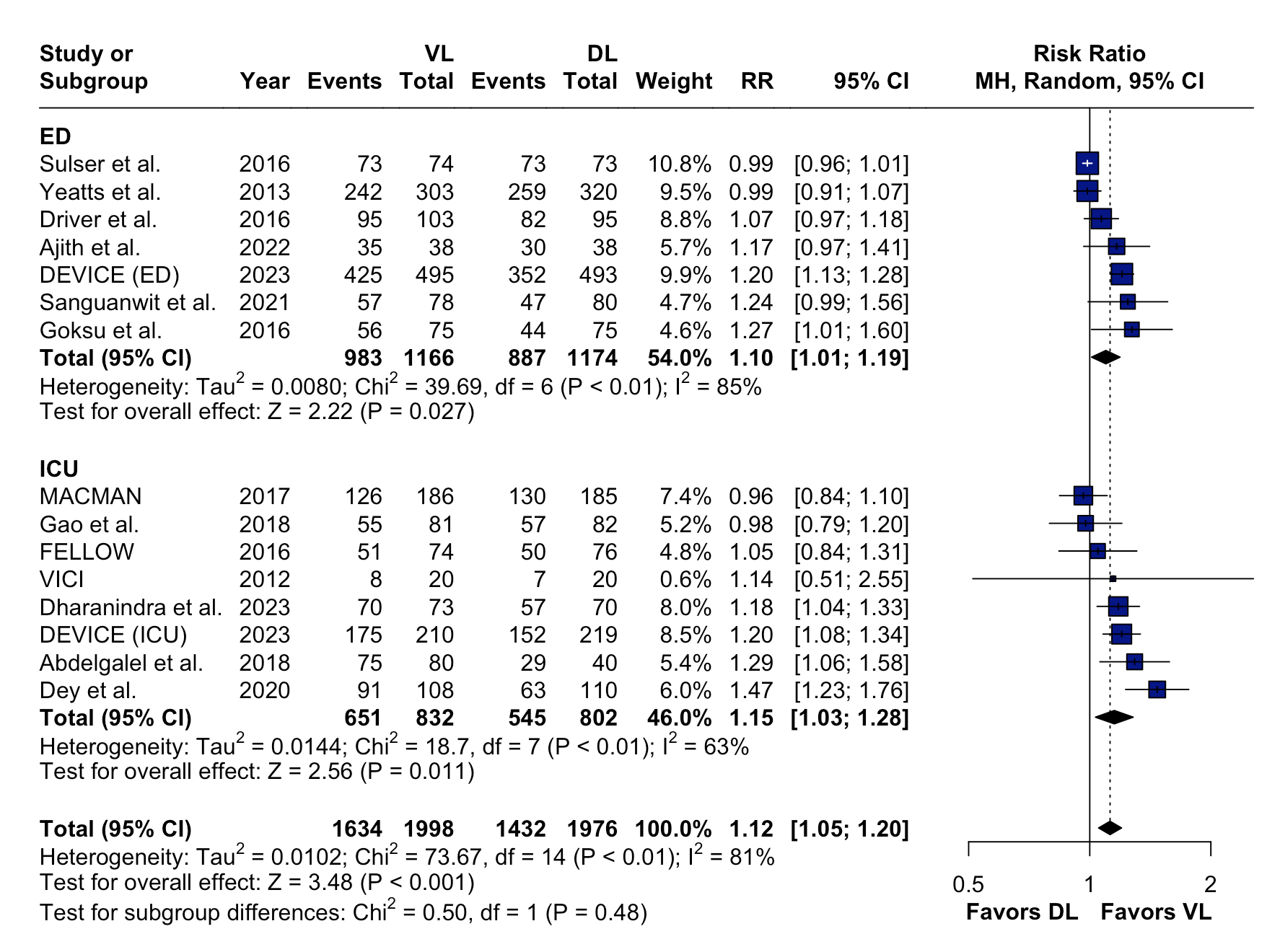
**

# Supplemental Figure 5. Subgroup Analysis Stratified by Operator’s Experience (Using a Threshold of 100 Prior Intubations)

**
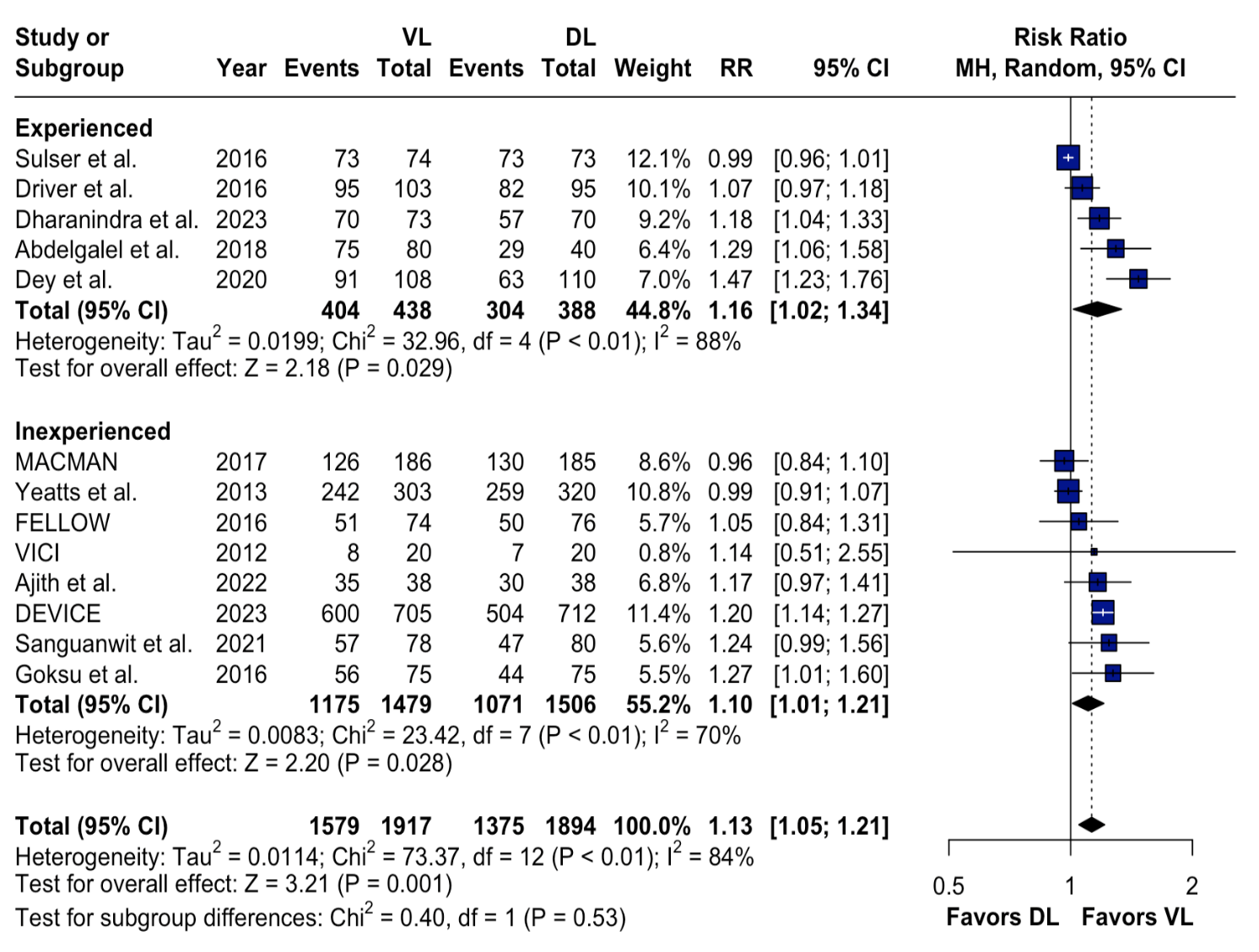
**

# Supplemental Figure 6. Graphical Display of Study Heterogeneity (GOSH)

**Figure 6A.** GOSH plot


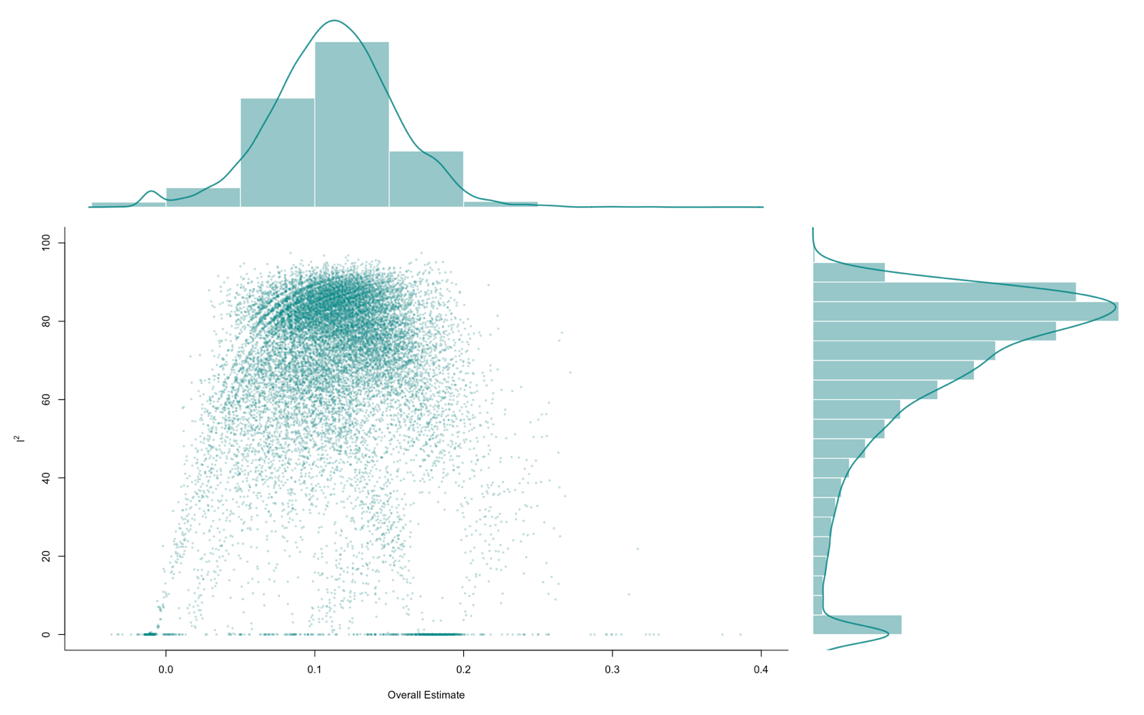


Legend: GOSH plot of I^2^ against summary effect sized (log risk ratio).

**Figure 6B.** K-means Algorithm


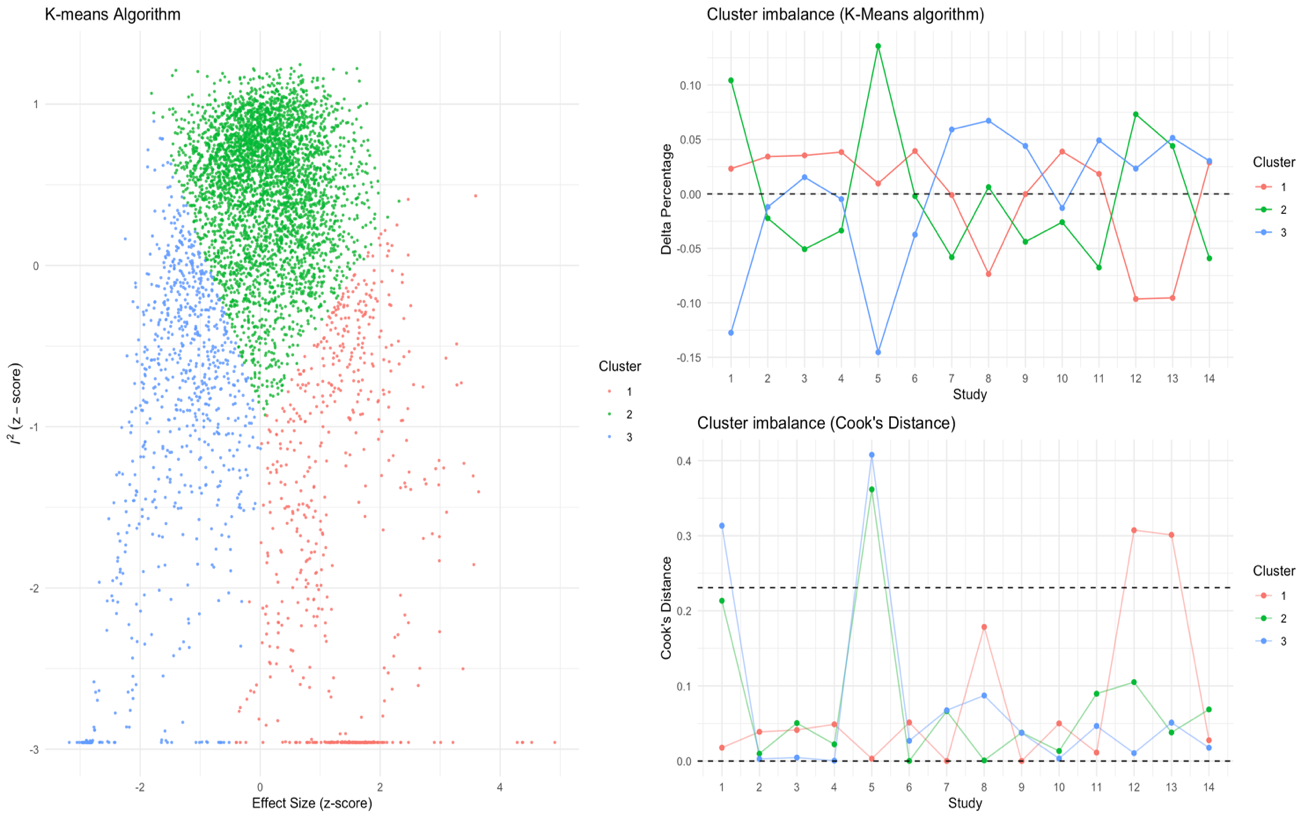


**Figure 6C.** Density-based spatial clustering of applications with noise (DBSCAN) Algorithm


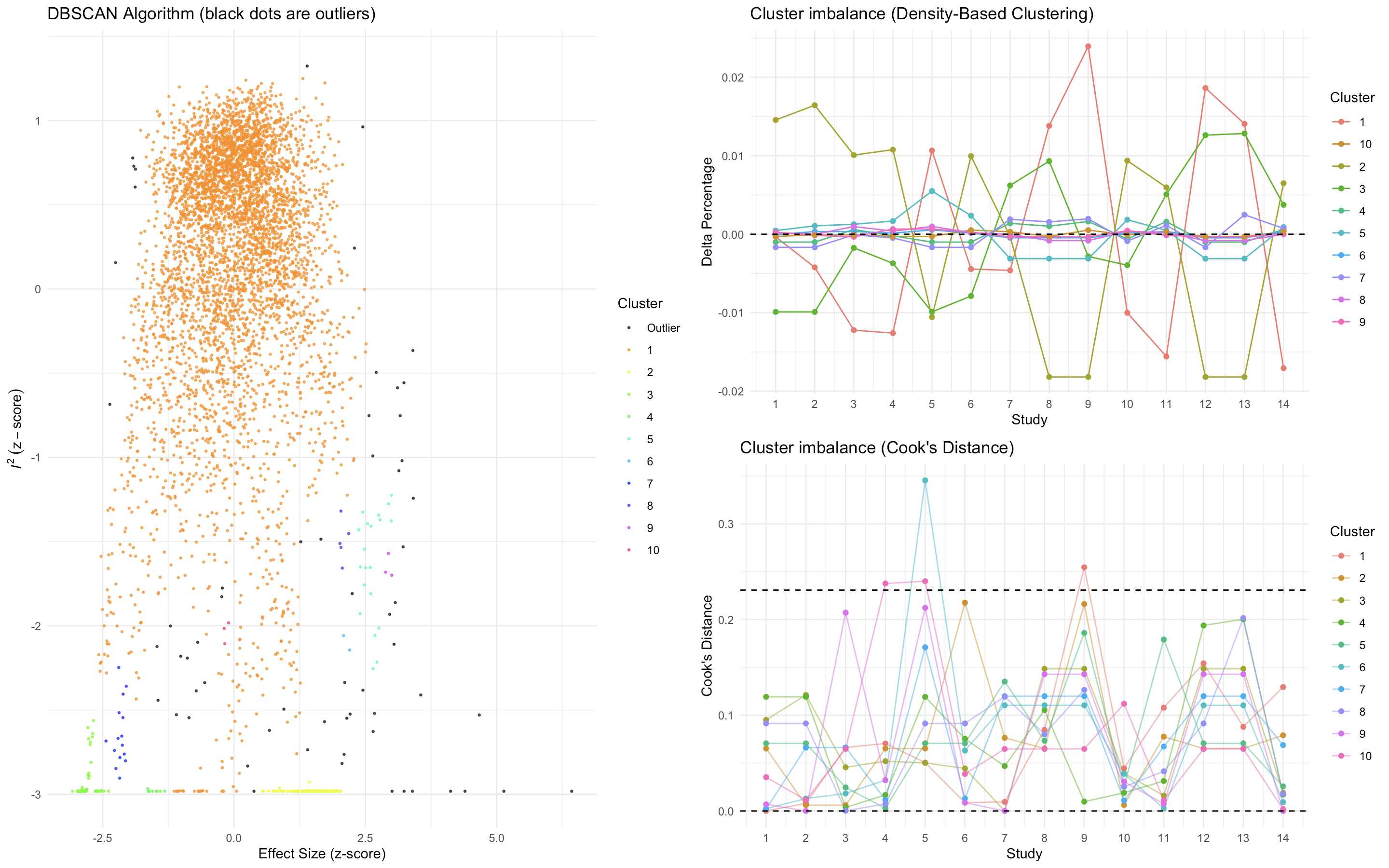


**Figure 6D.** Gaussian Mixture Model

**
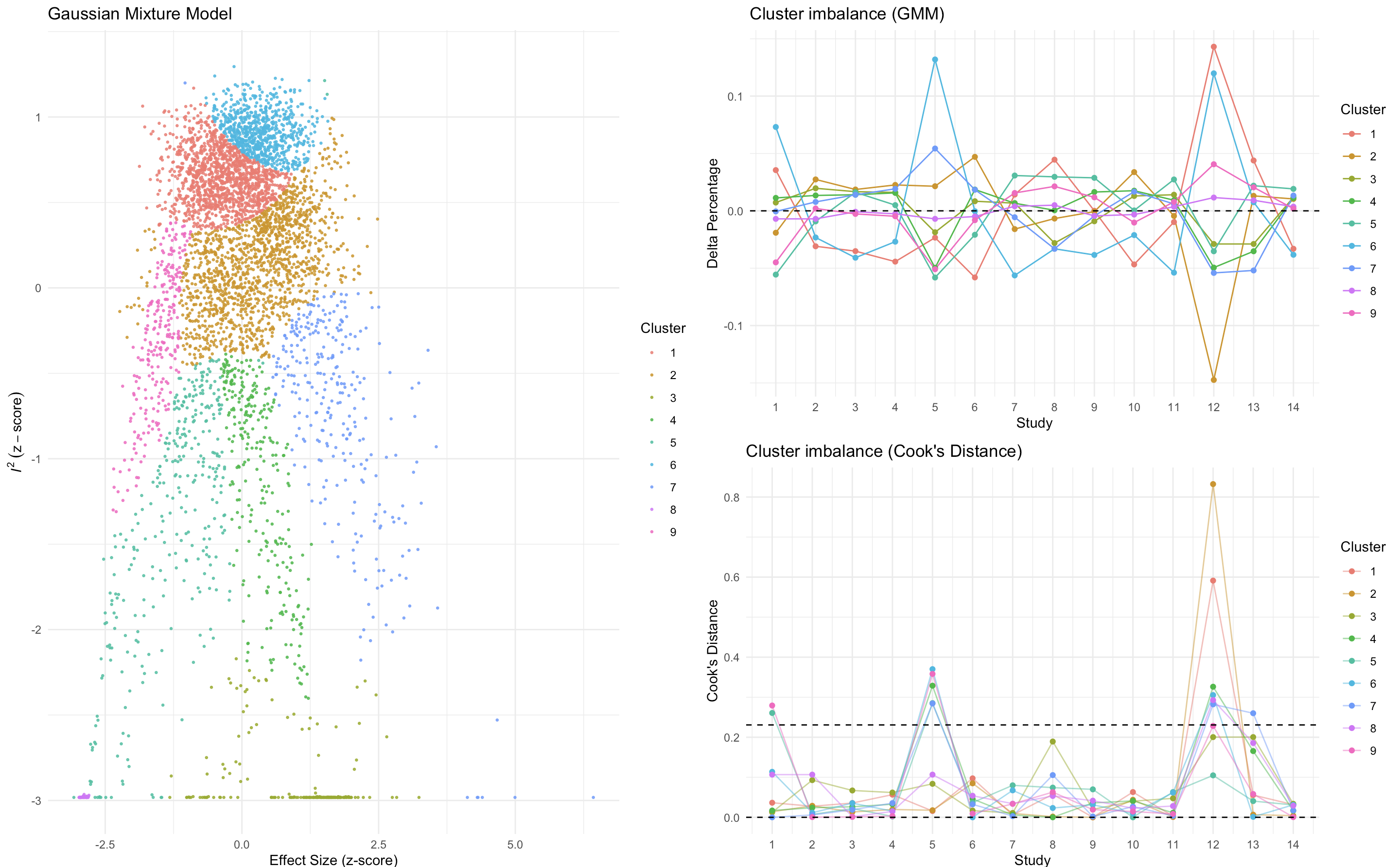
**

**Figure 6E.** GOSH Plot Analysis R Output for Primary Endpoint – Identification of Potential Outliers


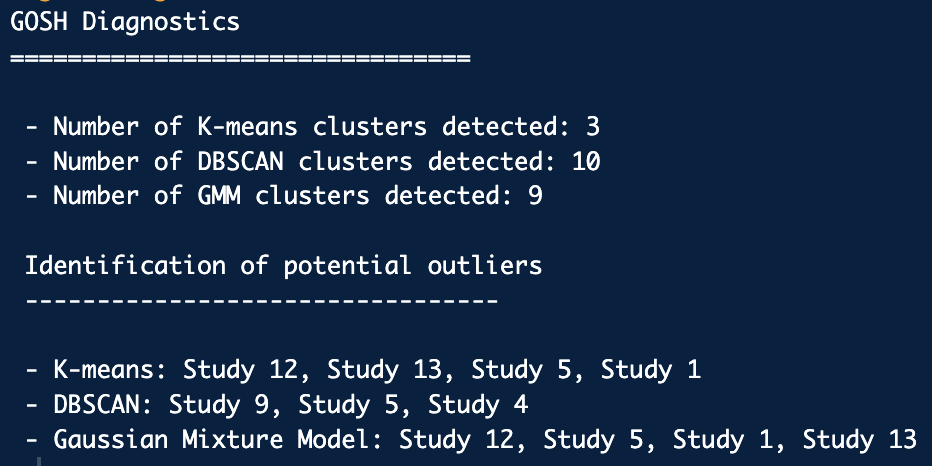


Study 12 = FELLOW 2016

Study 13 = Sulser et al., 2016

Study 5 = Dey et al., 2020*

Study 1 = DEVICE trial

*Study detected by all algorithm

**Figure 6F.** GOSH plots with the corresponding subset


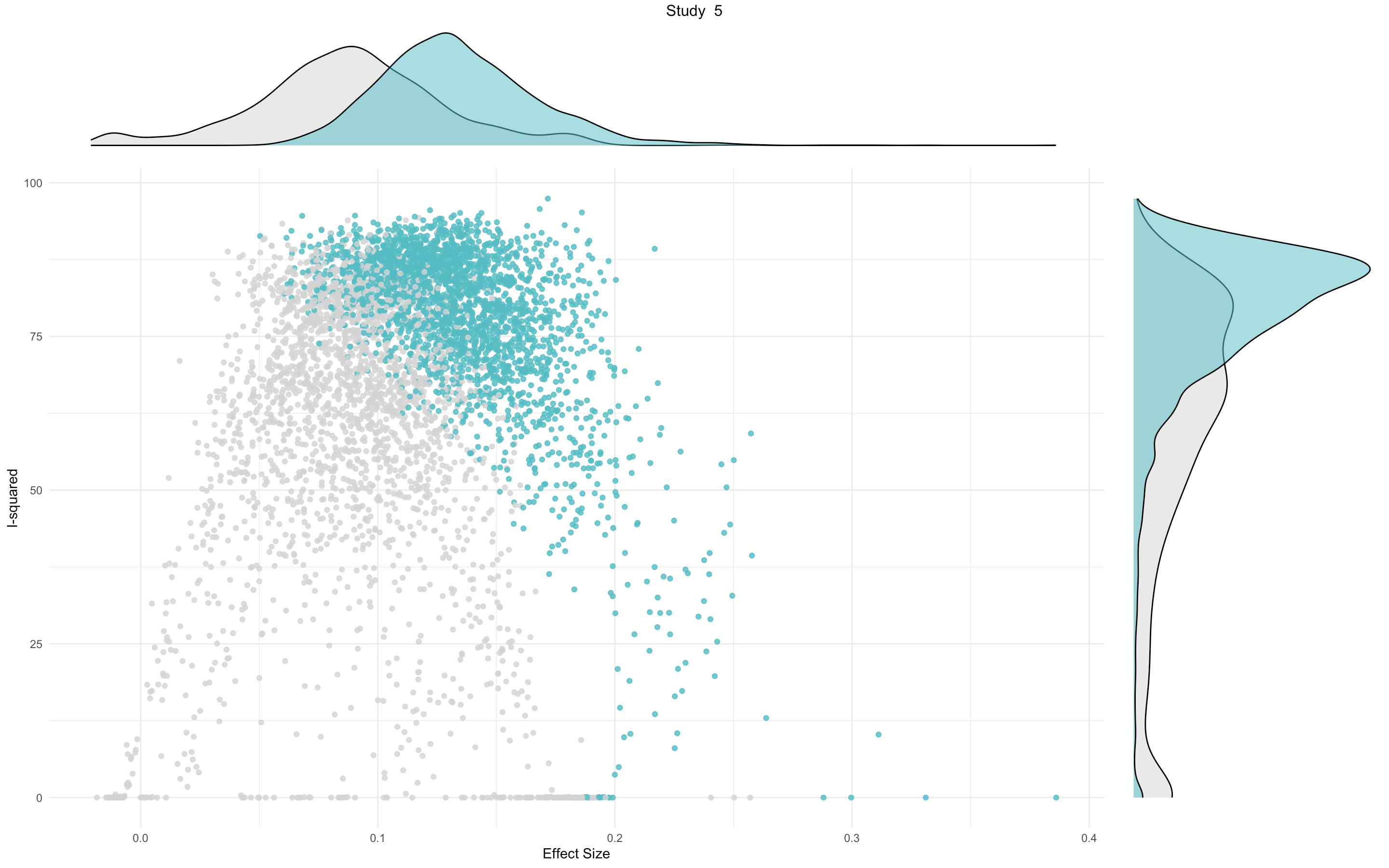


Legend: GOSH plots with the corresponding subset, including the potential outlier (Dey et al, 2020) colored in cyan.

# Supplemental Figure 7. Baujat Plot of the Primary Efficacy Endpoint


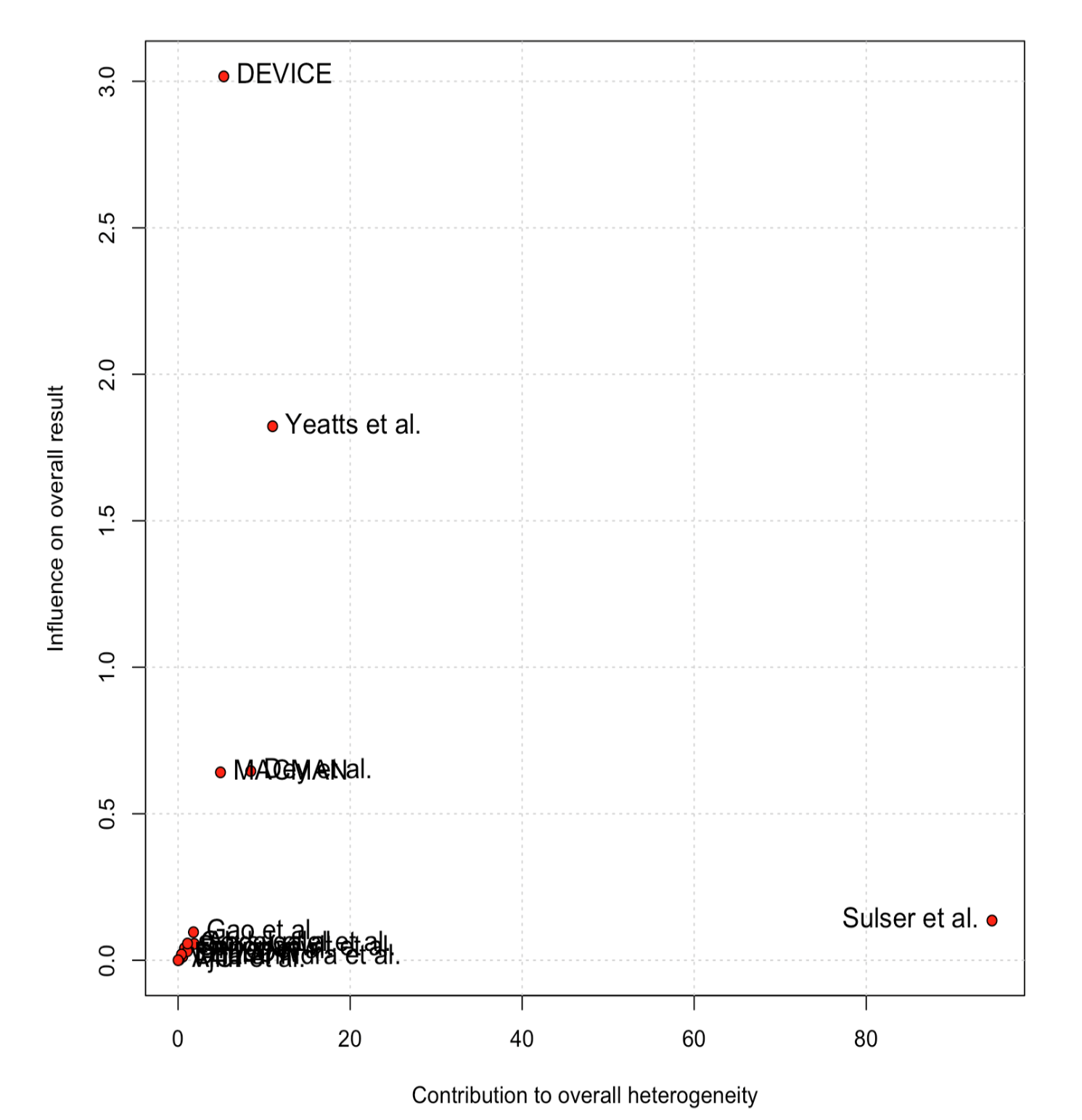


# Supplemental Figure 8. Leave-one-out Sensitivity Analysis of the Primary Efficacy Endpoint

**
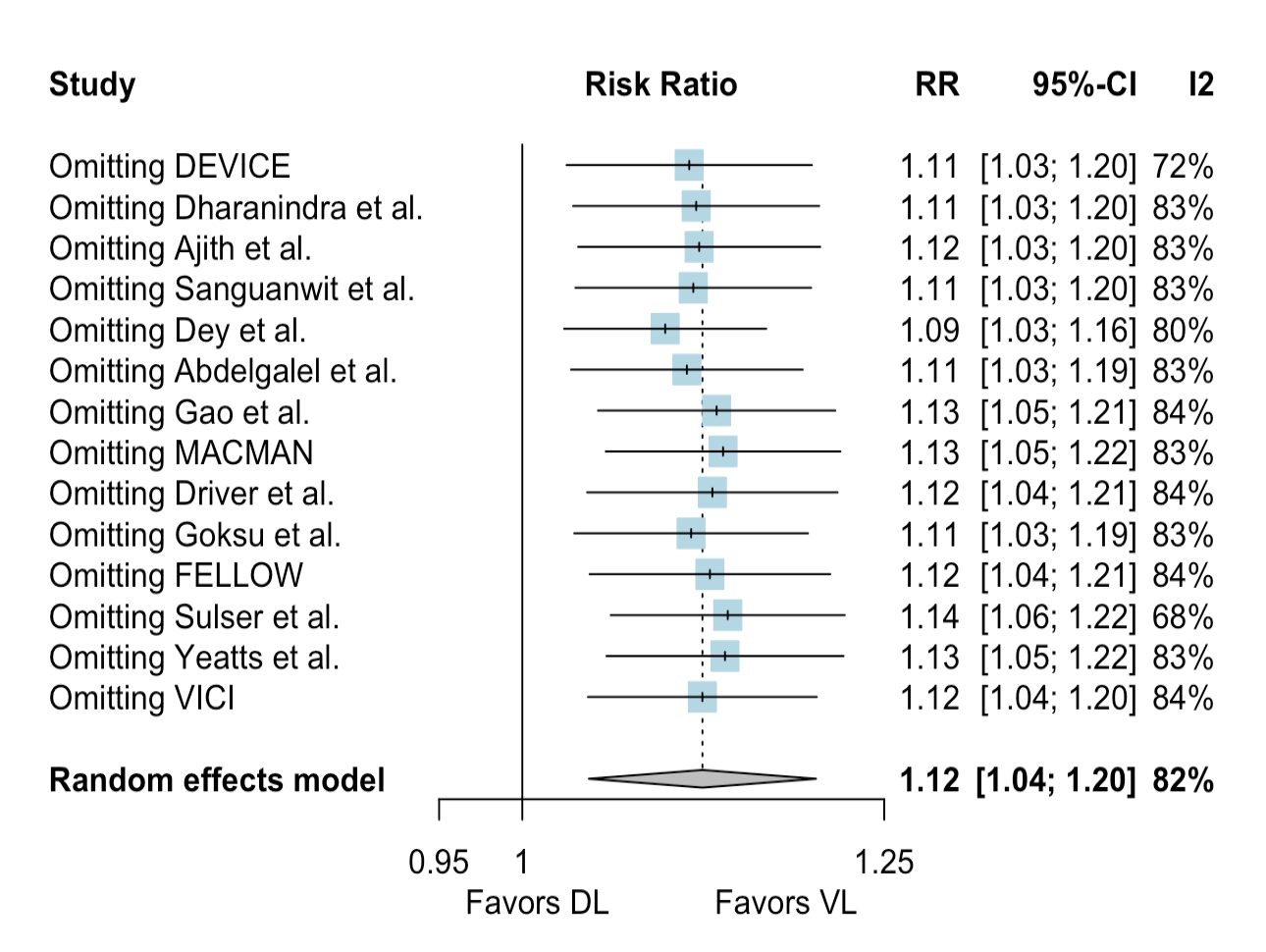
**

# Supplemental Figure 9. Risk of Bias 2 of All Included Studies


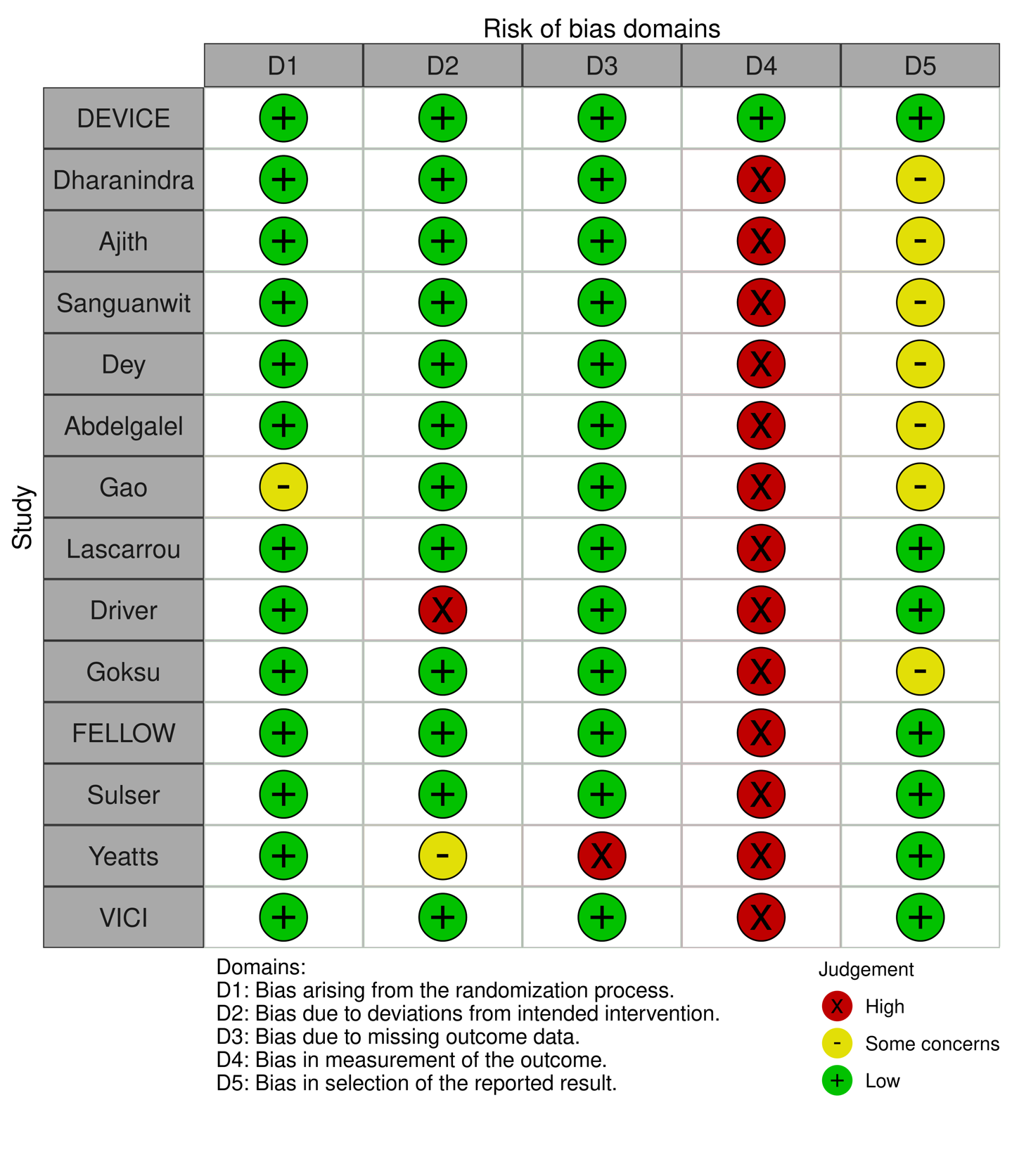


# Supplemental Figure 10. Funnel Plot and Egger’s Test for the Primary Efficacy Endpoint


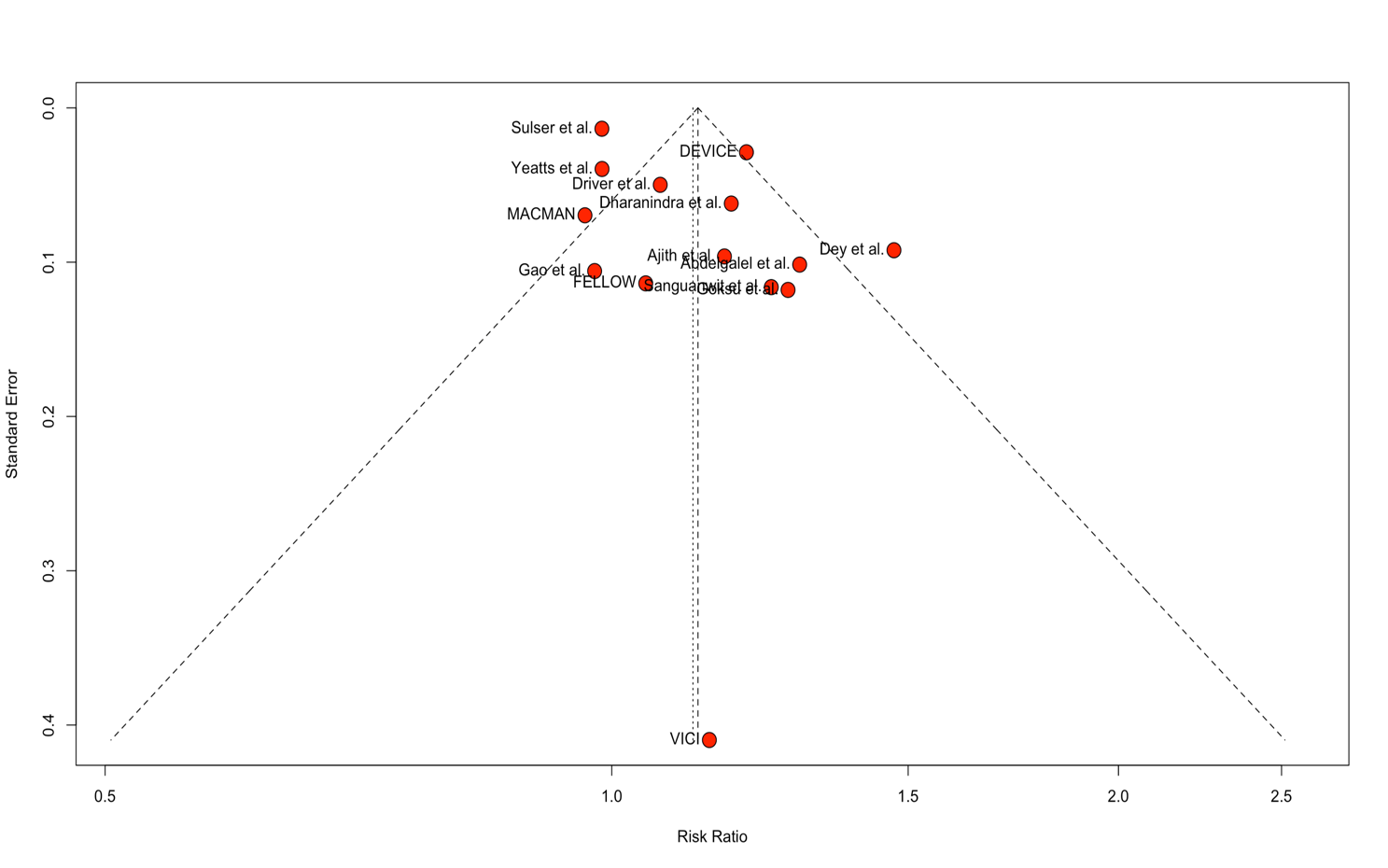


| **Egger’s Regression Test for Successful Intubation at First Attempt** | | | |
| --- | --- | --- | --- |
| **Intercept** | **95% CI** | **t** | **p-value** |
| 1.776 | 0.2 - 3.36 | 2.203 | 0.04787 |

# Supplemental Figures 11. Sensitivity Analyses of the Primary Outcome

**Figure 11A.** Sensitivity Analysis without the main outlier (detected by all algorithm)
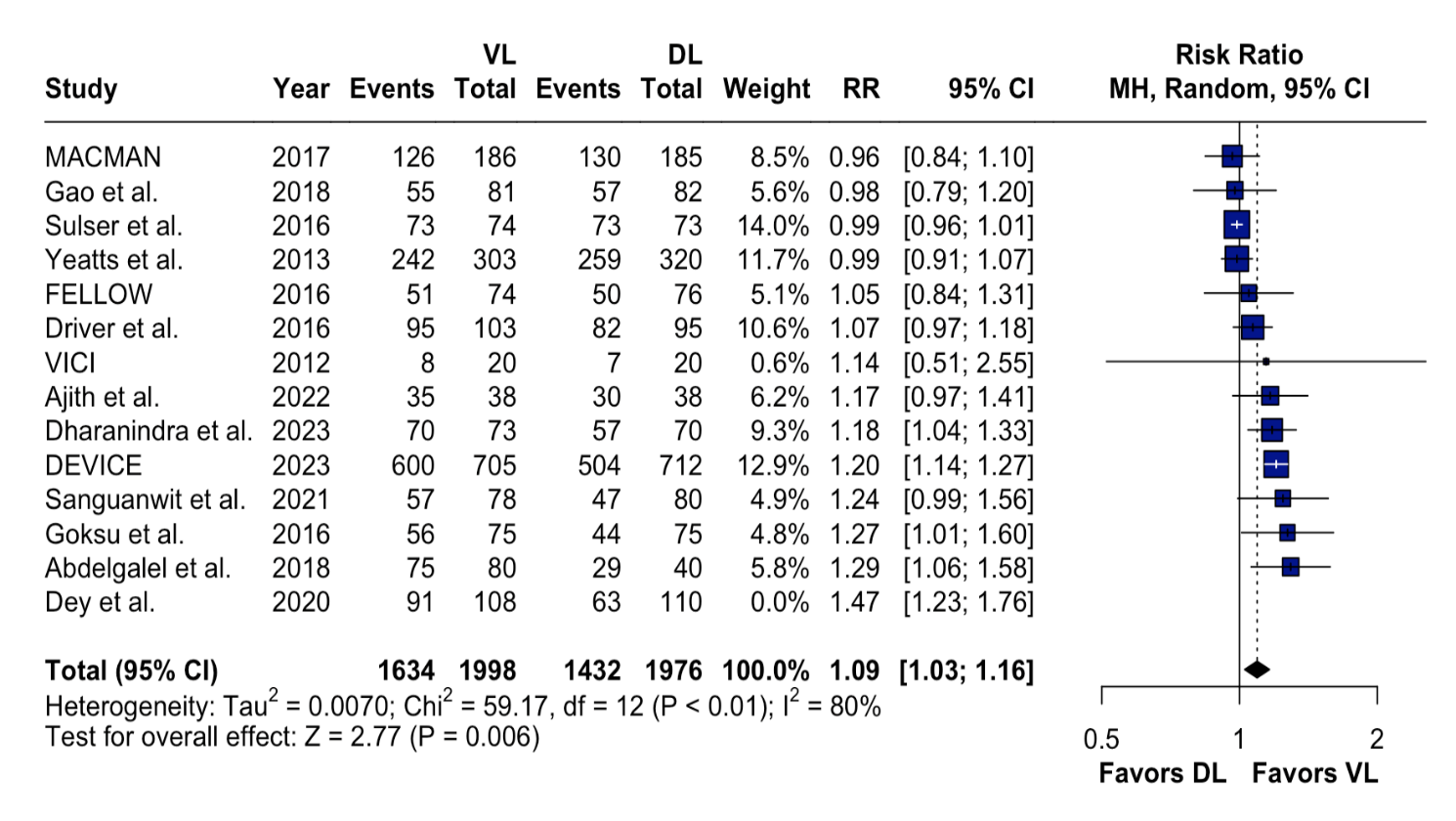


**Figure 11B.** Sensitivity Analysis without the main outliers (detected by at least 2 algorithm)


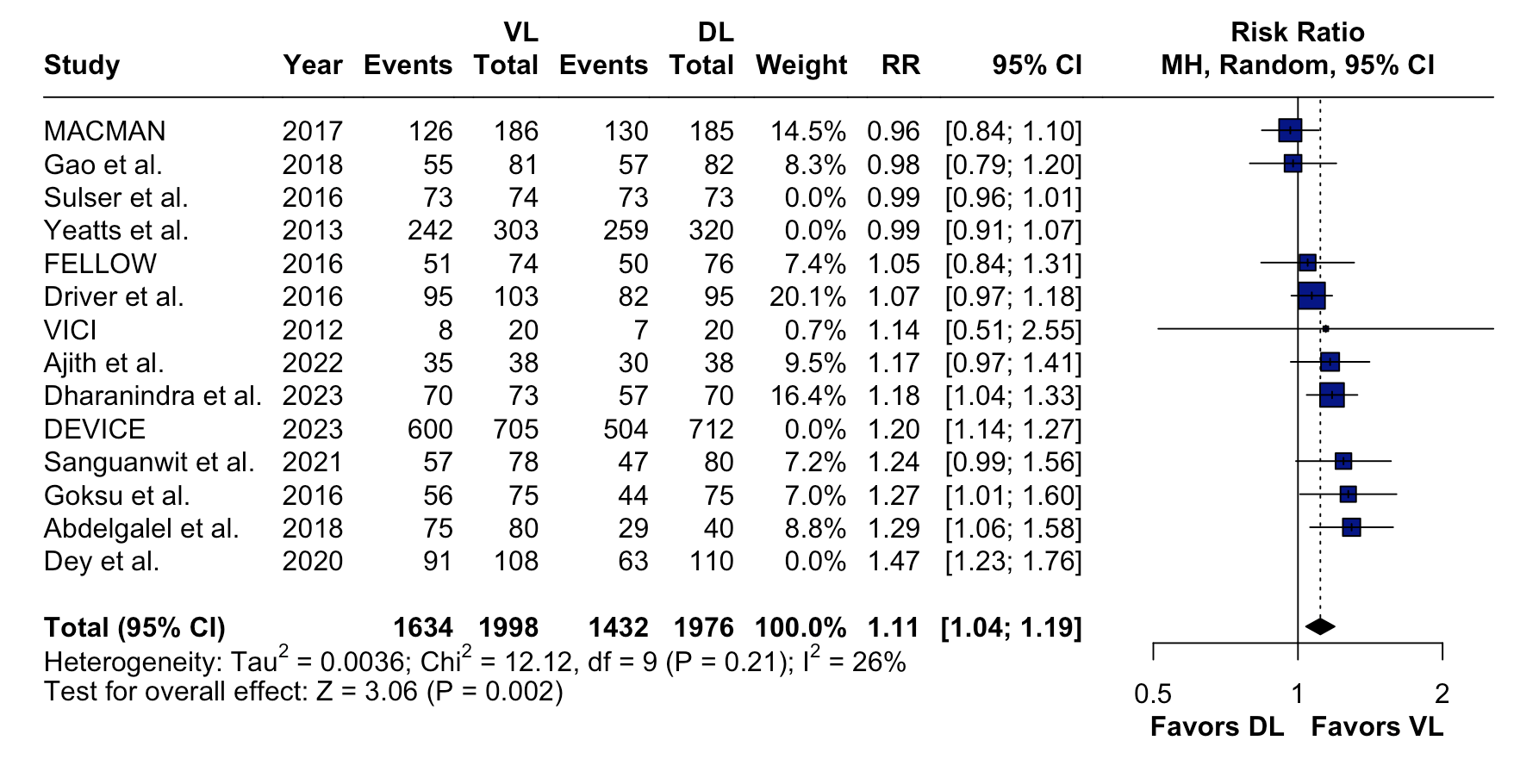


# Supplemental Figures 12. *Influence.Analysis* and *find.outliers* R Functions for the Primary Outcome

**Figure 12A.** *Influence.Analysis* **
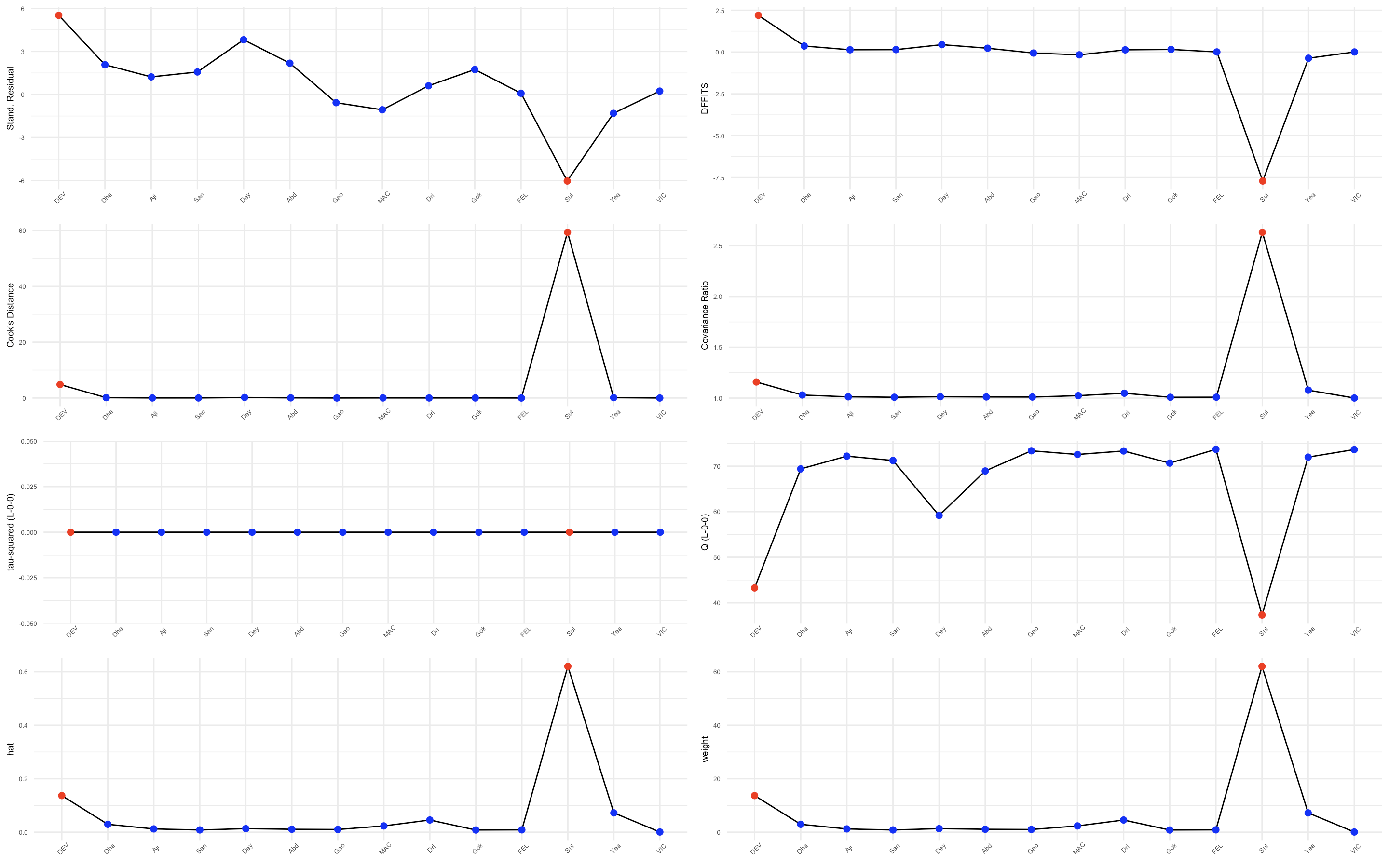
**

**Figure 12B.** *find.outliers*

**
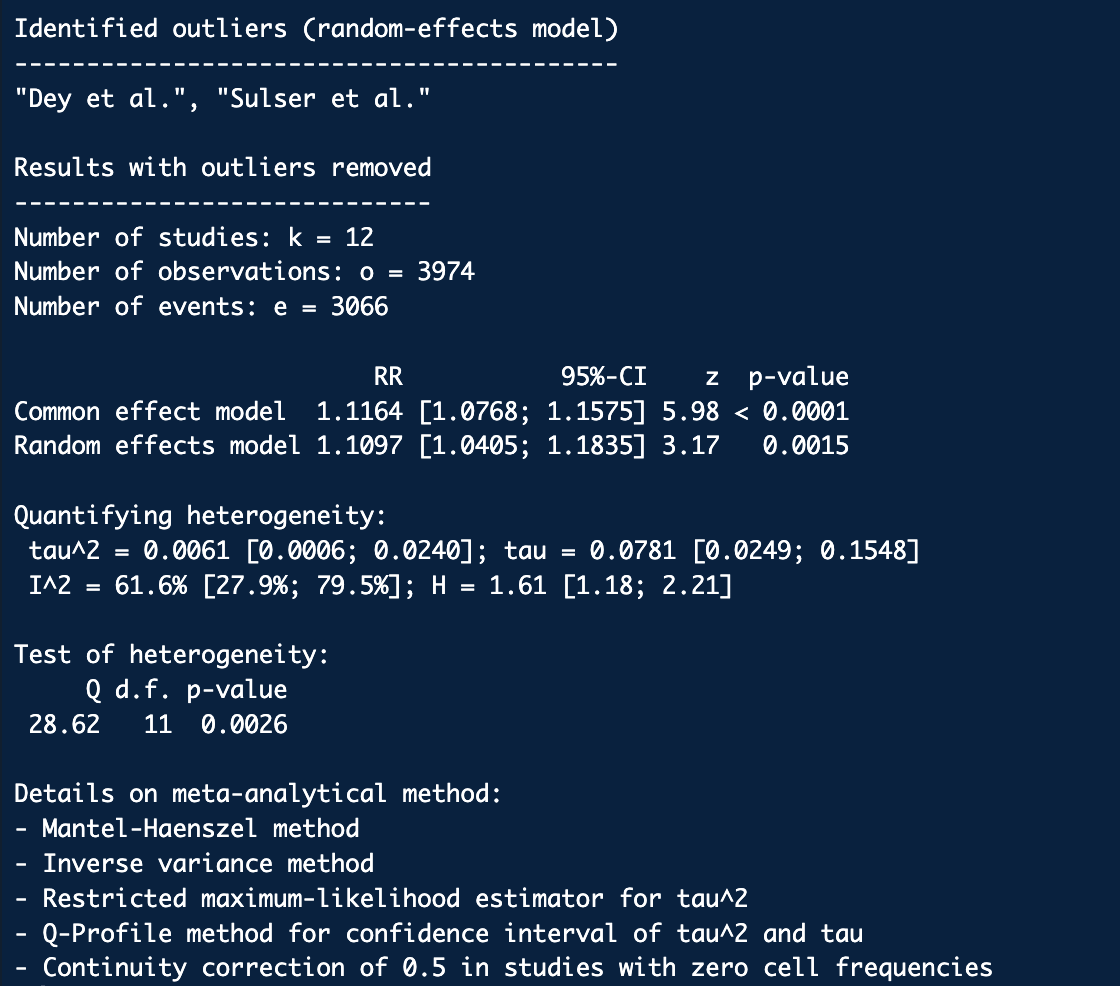
**

# Supplemental References

1. Olkin I, Dahabreh IJ, Trikalinos TA (2012) GOSH – a graphical display of study heterogeneity. Res Synth Methods 3:214–223. https://doi.org/10.1002/JRSM.1053

2. Hartigan JA, Wong MA (1979) A K-Means Clustering Algorithm. J R Stat Soc Ser C Appl Stat 28:100–108. https://doi.org/10.2307/2346830

3. Schubert E, Sander J, Ester M, et al (2017) DBSCAN Revisited, Revisited. ACM Transactions on Database Systems (TODS) 42:. https://doi.org/10.1145/3068335

4. Fraley C, Raftery AE (2002) Model-Based Clustering, Discriminant Analysis, and Density Estimation. J Am Stat Assoc 97:611–631. https://doi.org/10.1198/016214502760047131

5. Baujat B, Mahé C, Pignon JP, Hill C (2002) A graphical method for exploring heterogeneity in meta-analyses: Application to a meta-analysis of 65 trials. Stat Med 21:2641–2652. https://doi.org/10.1002/sim.1221

6. Viechtbauer W, Cheung MW-L (2010) Outlier and influence diagnostics for meta-analysis. Res Synth Methods 1:112–125. https://doi.org/10.1002/JRSM.11

7. Efthimiou O (2018) Practical guide to the meta-analysis of rare events. Evid Based Ment Health 21:72–76. https://doi.org/10.1136/EB-2018-102911

8. Sidik K, Jonkman JN (2007) A comparison of heterogeneity variance estimators in combining results of studies. Stat Med 26:1964–1981. https://doi.org/10.1002/SIM.2688

9. Sidik K, Jonkman JN (2002) A simple confidence interval for meta-analysis. Stat Med 21:3153–3159. https://doi.org/10.1002/SIM.1262

10. Knapp G, Hartung J (2003) Improved tests for a random effects meta-regression with a single covariate. Stat Med 22:2693–2710. https://doi.org/10.1002/SIM.1482

11. Higgins JPT, Thompson SG (2002) Quantifying heterogeneity in a meta-analysis. Stat Med 21:1539–1558. https://doi.org/10.1002/sim.1186

12. Mavridis D, Salanti G (2014) How to assess publication bias: funnel plot, trim-and-fill method and selection models. Evid Based Ment Health 17:30–30. https://doi.org/10.1136/EB-2013-101699

13. Viechtbauer W (2010) Conducting Meta-Analyses in R with the metafor Package. J Stat Softw 36:. https://doi.org/10.18637/jss.v036.i03

14. Harrer M, CP, FT, EDD (2019) dmetar: Companion R Package For The Guide “Doing Meta-Analysis in R”. R package version 0.0.9000. In: http://dmetar.protectlab.org/

15. Balduzzi S, Rücker G, Schwarzer G (2019) How to perform a meta-analysis with R: a practical tutorial. In: Evidence-Based Mental Health, 22nd ed. pp 153–160

16. Wickham H (2016) ggplot2: Elegant Graphics for Data Analysis. Springer New York, New York, NY
